# Immunomodulation by systemic administration of human-induced pluripotent stem cell-derived mesenchymal stromal cells to enhance the therapeutic efficacy of cell-based therapy for treatment of myocardial infarction

**DOI:** 10.7150/thno.46119

**Published:** 2021-01-01

**Authors:** Si-Jia Sun, Wing-Hon Lai, Yu Jiang, Zhe Zhen, Rui Wei, Qizhou Lian, Song-Yan Liao, Hung-Fat Tse

**Affiliations:** 1 Cardiology Division, Department of Medicine, Queen Mary Hospital, the University of Hong Kong, Hong Kong SAR, China.; 2 Shenzhen Institutes of Research and Innovation, the University of Hong Kong, Hong Kong SAR, China.; 3 Hong Kong-Guangdong Joint Laboratory on Stem Cell and Regenerative Medicine, the University of Hong Kong and Guangzhou Institutes of Biomedicine and Health, China.; 4 Department of Medicine, Shenzhen Hong Kong University Hospital, Shenzhen, China.

**Keywords:** human induced pluripotent stem cell, immunomodulation, mesenchymal stromal cell, cardiomyocyte, myocardial infarction

## Abstract

**Rationale:** Poor survival and engraftment are major hurdles of stem cell therapy in the treatment of myocardial infarction (MI). We sought to determine whether pre-transplantation systemic intravenous administration of human induced pluripotent stem cell (hiPSC)-derived mesenchymal stromal cells (hiPSC-MSCs) could improve the survival of hiPSC-MSCs or hiPSC-derived cardiomyocytes (hiPSC-CMs) following direct intramyocardial transplantation in a mouse model of MI.

**Methods:** Mice were randomized to undergo intravenous administration of saline or 5×10^5^ hiPSC-MSCs one week prior to MI, induced by ligation of the left anterior descending coronary artery. Mice were further assigned to undergo direct intramyocardial transplantation of hiPSC-MSCs (1×10^6^) or hiPSC-CMs (1×10^6^) 10 minutes following MI. Echocardiographic and invasive hemodynamic assessment were performed to determine cardiac function. *In-vivo* fluorescent imaging analysis, immunofluorescence staining and polymerase chain reaction were performed to detect cell engraftment. Flow cytometry of splenic regulatory T cells (Tregs) and natural killer (NK) cells was performed to assess the immunomodulatory effects.

**Results:** Pre-transplantation systemic administration of hiPSC-MSCs increased systemic Tregs activation, decreased the number of splenic NK cells and inflammation, and enhanced survival of transplanted hiPSC-MSCs and hiPSC-CMs. These improvements were associated with increased neovascularization and decreased myocardial inflammation and apoptosis at the peri-infract zone with consequent improved left ventricular function four weeks later. Co-culture of splenic CD4 cells with hiPSC-MSCs also modulated their cytokine expression profile with a decreased level of interferon-γ, tumor necrosis factor-α, and interleukin (IL)-17A, but not IL-2, IL-6 and IL-10.

**Conclusion:** Pre-transplantation systemic intravenous administration of hiPSC-MSCs induced immunomodulation and facilitated the survival of intramyocardially transplanted cells to improve cardiac function in MI.

## Introduction

Despite recent advances in reperfusion therapy and pharmacotherapy for myocardial infarction (MI), a significant proportion of patients develop intractable heart failure (HF) due to progressive left ventricular (LV) remodeling 1, 2. Different cell-based therapies to replenish the loss of cardiomyocytes (CMs) have been investigated for treatment of MI 3. Unfortunately, clinical 4-6 and preclinical trials 7, 8 that focused on the transplantation of autologous somatic cells, such as bone marrow (BM) cells, failed to demonstrate any significant clinical benefits. One of the major hurdles is the inconsistent number and quality of autologous stem cells that leads to discordance in clinical outcome 9. Even allogenic somatic cell sources from healthy donors, such as mesenchymal stromal cells (MSCs) derived from BM and adipose tissue, are limited by their senescence after several passages and problems with standardization and batch-to-batch variation with different donors 10, 11.

Recently, our group has generated MSCs from human-induced pluripotent stem cells (hiPSCs) with enhanced proliferation and immune tolerance capacities. This offers an unlimited “*off-the-shelf*” cell source with predictable therapeutic efficacy 12, 13. We have demonstrated that hiPSC-MSCs have better proliferative capacity, survival and therapeutic efficacy for myocardial repair than BM-MSCs 12 or embryonic stem cell-derived CMs 14. Compared with BM-MSCs, hiPSC-MSCs are more immune-privileged due to their insensitivity to pro-inflammatory interferon*-γ-*induced human leukocyte antigen (HLA) class II expression 15. In addition, hiPSC-MSCs possess multiple immunomodulatory actions similar to BM-MSCs, suppressing proliferation, cytokine secretion and cytotoxicity of immune T-cells; and modulating the functions of regulatory T cells (Tregs) and natural killer (NK) cells 15, 16. Nevertheless, local myocardial delivery of hiPSC-MSCs may not induce these systemic immunomodulatory effects so their survival in the infarcted myocardium remains poor 14, 17. Currently, the main strategy to improve survival of locally transplanted cells is to use long-term immunosuppressive agents but this is associated with toxicity and adverse effects 18, 19. As a result, systemic administration of MSCs has been explored as an immunomodulation therapy for graft-versus-host-disease following BM transplantation 18, as well to improve graft survival following solid organ transplantation 19.

We hypothesized that pre-transplantation systemic administration of hiPSC-MSCs would induce immunomodulation and enhance the engraftment and therapeutic effects of intramyocardial implantation of cells after MI. The potential immunomodulatory ability of hiPSC-MSCs to enhance the survival and engraftment of different types of transplanted cells using hiPSC-MSCs or hiPSC-CMs was also investigated.

## Methods

**Figure 1:**
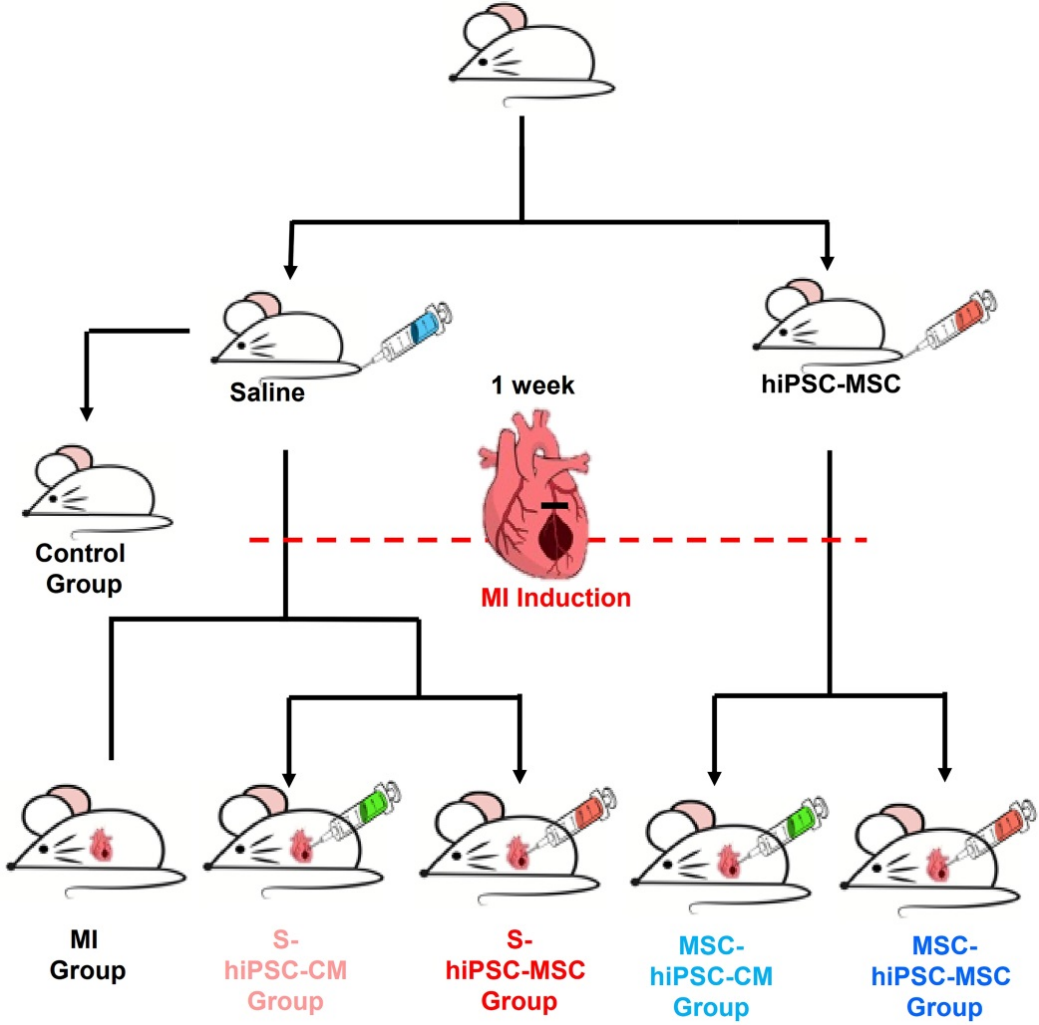
Flow chart of the experiment.

The study design is outlined in **Figure 1**; full details of the reagents and experimental procedures are described in the **Online Supplemental Methods.**

### Statistical analysis

All data are expressed as mean ± SEM, and analysis was performed using SPSS software (SPSS, Inc., Chicago, IL, USA). The Student t test was used to compare two groups. Comparison of variables between multiple groups was performed using one-way ANOVA with Tukey post hoc test. All data were analyzed in a blind manner. A *P* value ≤ 0.05 was considered statistically significant.

## Results

As shown in **Figure 1**, 88 mice were randomized to one of six groups: control group (n=8), MI group (n=10), S-hiPSC-CM group (n=17), S-hiPSC-MSC group (n=17), MSC-hiPSC-CM group (n=18), and MSC-hiPSC-MSC group (n=18). 2 mice each from the MI group and S-hiPSC-MSC group, and 3 mice each from the S-hiPSC-CM, MSC-hiPSC-CM and MSC-hiPSC-MSC group died after induction of MI. In addition, 1 mouse from the S-hiPSC-MSC group and 1 mouse from MSC-hiPSC-CM group were excluded as there was no evidence of MI (determined by Masson Trichrome staining). After intramyocardial cell transplantation, 3 mice each from S-hiPSC-CM, MSC-hiPSC-CM, S-hiPSC-MSC and MSC-hiPSC-MSC group were sacrificed immediately for fluorescent imaging analysis of DiR signal. Then, a total of 12 mice from the S-hiPSC-CM group (n=3), S-hiPSC-MSC group (n=3), MSC-hiPSC-CM group (n=3) and MSC-hiPSC-MSC group (n=3) were sacrificed on day 7 for fluorescent imaging analysis of DiR signal. As a result, 49 mice completed this study and were sacrificed on day 28: eight mice each from the control, MI, S-hiPSC-CM, S-hiPSC-MSC and MSC-hiPSC-CM groups and nine mice from the MSC-hiPSC-MSC group.

### Improvement in LV function after transplantation

**Figure 2:**
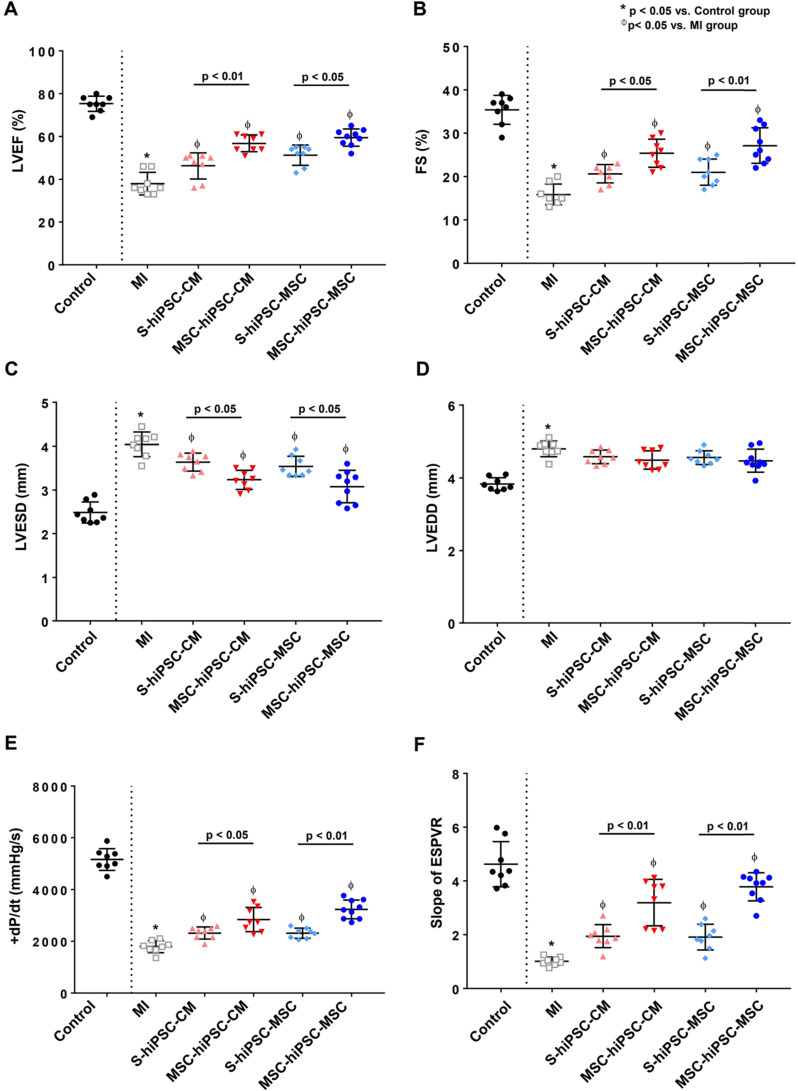
** Intravenous pre-transplantation systemic administration of hiPSC-MSCs improved left ventricular function.** To assess left ventricular (LV) function, echocardiographic measurement was performed of left ventricular ejection fraction (LVEF) **(A)**, fractional shortening (FS) **(B)**, LV end-systolic dimension (LVESD) **(C)**, and LV end-diastolic dimension (LVEDD) **(D)**. Intravenous pre-transplantation of hiPSC-MSCs could further improve LVEF, increase FS and decrease LVESD in the MSC-hiPSC-CM group and MSC-hiPSC-MSC group compared with the S-hiPSC-CM group and S-hiPSC-MSC group. No change was observed in LVEDD; invasive hemodynamic study was performed to determine the maximal positive pressure derivative (+dP/dt_max_) **(E)**, and the slope of end systolic pressure-volume relationship (ESPVR) **(F)** 4 weeks following induction of myocardial infarction (MI) in different groups of animals. Intravenous pre-transplantation of hiPSC-MSCs could further improve +dP/dt_max_ and ESPVR in MSC-hiPSC-CM group and MSC-hiPSC-MSC group compared with S-hiPSC-CM group and S-hiPSC-MSC group.

Transthoracic echocardiogram was performed to measure LV ejection fraction (LVEF), fractional shortening (FS) and LV dimension (**Figure S1A**). Compared with the control group, LVEF (75.3±1.3% versus 37.9±1.9%; *P*<0.01*)* and FS (35.4±1.2% versus 15.9±0.9%; *P*<0.01) were decreased (**Figure 2A-B**) and LV end-systolic dimension (LVESD) (2.5±0.1% versus 4.0±0.1%; *P*<0.01) was increased in the MI group (**Figure 2C**). Compared with the MI group, LVEF and FS were significantly increased (**Figure 2A-B**; *P*<0.05) and LVESD was significantly decreased (**Figure 2C**; *P<0.05*) in S-hiPSC-CM, MSC-hiPSC-CM, S-hiPSC-MSC and MSC-hiPSC-MSC groups. Moreover, LVEF and FS were further increased (**Figure 2A-B**; *P*<0.05); and the LVESD were decreased (**Figure 2C**; *P*<0.05) in the MSC-hiPSC-CM and MSC-hiPSC-MSC compared with the S-hiPSC-CM and S-hiPSC-MSC groups, respectively after transplantation. Nevertheless, there was no difference in LV end-diastolic dimension (LVEDD) between MI group and all treatment groups (**Figure 2D**; *P*>0.05).

Invasive hemodynamic assessment of pressure-volume loop was performed to measure LV maximal positive pressure derivative (+dP/dt_max_) and the end-systolic pressure-volume relationship (ESPVR) (**Figure S1B**). Compared with the control group, +dP/dt_max_ (+5164±148 mmHg/s versus +1793±83 mmHg/s; *P*<0.01) and ESPVR (4.6±0.3 versus 1.0±0.1; *P*<0.01) were decreased in the MI group (**Figure 2E-F**). Compared with the MI group, +dP/dt_max_ and ESPVR were significantly increased in S-hiPSC-CM, MSC-hiPSC-CM, S-hiPSC-MSC and MSC-hiPSC-MSC groups (**Figure 2E-F**; *P*<0.05). Moreover, +dP/dt_max_ (**Figure 2E**; *P*<0.05) and ESPVR (**Figure 2F**; *P*<0.01) were further increased in both MSC-hiPSC-CM and MSC-hiPSC-MSC groups compared with S-hiPSC-CM and S-hiPSC-MSC groups, respectively after transplantation. Our results showed that pre-transplantation systemic administration of hiPSC-MSCs enhanced the therapeutic benefit of intramyocardial transplantation of both hiPSC-CMs and hiPSC-MSCs in improving LV function post-MI.

### Changes in infarct size after transplantation

Masson trichrome staining was performed to assess infarct size 4 weeks after transplantation (**Figure S2A**). Compared with the MI group, the infarct size was significantly reduced in S-hiPSC-CM, MSC-hiPSC-CM, S-hiPSC-MSC and MSC-hiPSC-MSC groups (**Figure S2B**; *P*<0.05). Nevertheless, there were no significant differences in the infarct size in both MSC-hiPSC-CM and MSC-hiPSC-MSC groups did not further decrease infarct size compared with the S-hiPSC-CM and S-hiPSC-MSC groups, respectively (**Figure S2B**; *P>*0.0*5*). We observed no tumor formation at the injection site or other sites over the myocardium or other organs.

### Improved engraftment and survival after transplantation

**Figure 3:**
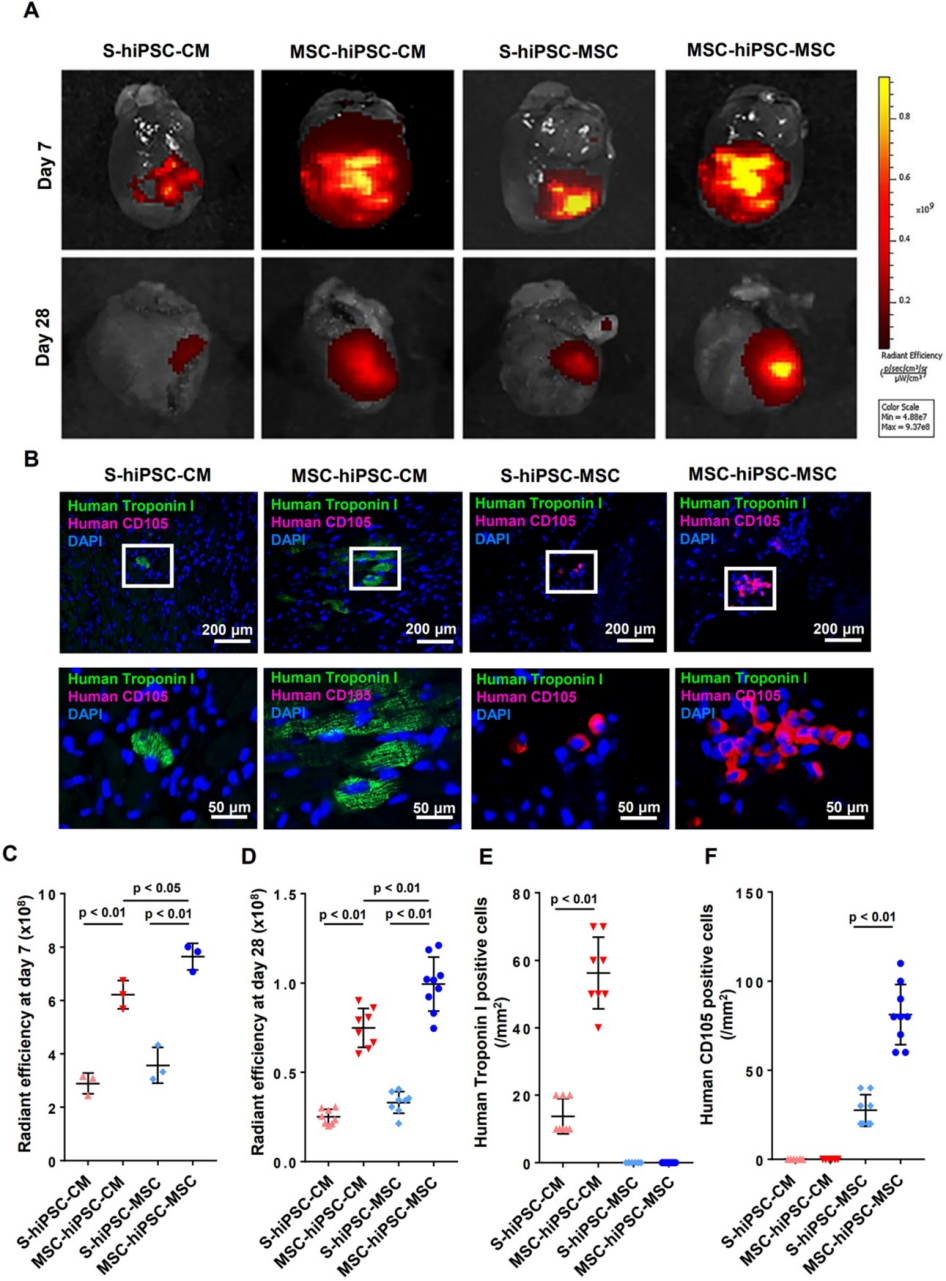
** Intravenous pre-transplantation systemic administration of hiPSC-MSCs improved survival of transplanted cells in mice with MI.** To evaluate the survival of intramyocardial transplanted hiPSC-cardiomyocytes (CMs) or hiPSC-MSCs, DiR labelled hiPSC-CMs or hiPSC-MSCs were detected on the infarcted hearts using an IVIS spectrum *in vivo* imaging system 7 and 28 days after intramyocardial transplantation (**A**). The quantity of intramyocardial transplanted hiPSC-MSCs and hiPSC-CMs in DiR signal images was indicated by the radiant efficiency of fluorescent intensity. The DiR signal was stronger in the MSC-hiPSC-CM group and MSC-hiPSC-MSC group compared with the S-hiPSC-CM group and S-hiPSC-MSC group respectively (**C-D**). Immunofluorescent staining of intramyocardial transplanted cells in the peri-infarct area was performed 4 weeks after induction of MI (**B**). The quantity of intramyocardial transplanted hiPSC-CMs and hiPSC-MSCs on immunofluorescent images was represented by the number of anti-human Troponin I positive cells and anti-human CD105 positive cells, respectively. Intramyocardially transplanted CMs were represented by human Troponin I positive cells (green). Intramyocardially transplanted MSCs were represented by human CD105 positive cells (red). Cell nuclei were counterstained with DAPI (blue). The quantity of human Troponin I positive cells and human CD105 positive cells were counted under fluorescent microscopy and expressed as count per mm^2^. The cell retention of transplanted hiPSC-CMs was increased in the MSC-hiPSC-CM group compared with the S-hiPSC-CM group (**E**). The cell retention of transplanted hiPSC-MSCs was increased in the MSC-hiPSC-MSC group compared with the S-hiPSC-MSC group (**F**). As no human CD105 positive cells were detected in the MSC-hiPSC-CM group, any human CD105 positive cells in the MSC-hiPSC-MSC group were considered to have derived from intramyocardially injected hiPSC-MSCs.

Fluorescent imaging of the harvested hearts was performed on day 0, day 7 and day 28 to evaluate cellular engraftment of the DiR-labeled hiPSC-CMs and hiPSC-MSCs at the peri-infarct zone of the LV (**Figure S3A & 3A**). There was no significant difference in fluorescent signal intensity over the LV on day 0 (**Figure S3B**). Pre-transplantation systemic administration of hiPSC-MSCs resulted in a significantly increased fluorescent signal intensity over the LV on day 7 or day 28 in the MSC-hiPSC-CM group and in the MSC-hiPSC-MSC group (**Figure 3C-D**; *P*<0.01) compared with the S-hiPSC-CM and S-hiPSC-MSC groups, respectively. Fluorescent signal intensity over the LV was also significantly higher in the MSC-hiPSC-MSC group compared with the MSC-hiPSC-CM group on day 7 (**Figure 3C**; *P*<0.05) and day 28 (**Figure 3D**; *P*<0.01). On the contrary, there was no difference between the S-hiPSC-MSC group and S-hiPSC-CM group on day 7(**Figure 3C**; *P*>0.05) or day 28 (**Figure 3D**; *P*>0.05). As shown in **Figure S3B,** the estimated survival rates of transplanted cells after intramyocardial injection at day 7 in S-hiPSC-CM, MSC-hiPSC-CM, S-hiPSC-MSC and MSC-hiPSC-MSC groups were 15.51%, 29.15%, 17.54% and 38.01%, respectively. At 28 days, the estimated survival rates of intramyocardial transplanted cells in S-hiPSC-CM, MSC-hiPSC-CM, S-hiPSC-MSC and MSC-hiPSC-MSC groups decreased to 1.35%, 3.51%, 1.62% and 4.95%, respectively.

Immunohistochemical analysis was performed to quantify cell engraftment of intramyocardially transplanted hiPSC-CMs or hiPSC-MSCs (**Figure 3B**). Since no human CD105 positive cells were detected in the MSC-hiPSC-CM group, any human CD105 positive cells in the MSC-hiPSC-MSC group were considered to have derived from intramyocardially injected hiPSC-MSCs. Pre-transplantation systemic administration of hiPSC-MSCs in both MSC-hiPSC-CM and MSC-hiPSC-MSC groups significantly increased the number of transplanted cells over the LV compared with the S-hiPSC-CM (**Figure 3E**; *P*<0.05) and S-hiPSC-MSC groups (**Figure 3F**; *P*<0.05), respectively.

Polymerase chain reaction (PCR) analysis of human DNA was also performed to determine the engraftment of transplanted cells. Although mouse mitochondrial DNA was detected in all groups, human GAPDH DNA was detected only in the four groups with cellular transplantation, not the MI group (**Figure S4A**). Qualitative PCR revealed that expression of the human GAPDH gene was significantly higher after pre-transplantation systemic administration of hiPSC-MSCs in both MSC-hiPSC-CM and MSC-hiPSC-MSC groups compared with the S-hiPSC-CM and S-hiPSC-MSC groups, respectively* (***Figure S4B**;* P*<0.01). Expression of human GAPDH was also significantly higher in the MSC-hiPSC-MSC group compared with the MSC-hiPSC-CM group (*P*<0.01). On the contrary, there was no difference between the S-hiPSC-MSC group and S-hiPSC-CM group (**Figure S4B**; *P*>0.05).

Our results showed that pre-transplantation systemic administration of hiPSC-MSCs enhanced hiPSC-CMs and hiPSC-MSCs engraftment and survival after intramyocardial transplantation although the benefit was greater for hiPSC-MSCs.

### Decreased immune cellular infiltration, cardiomyocyte hypertrophy and apoptosis in the peri-infarct area

**Figure 4:**
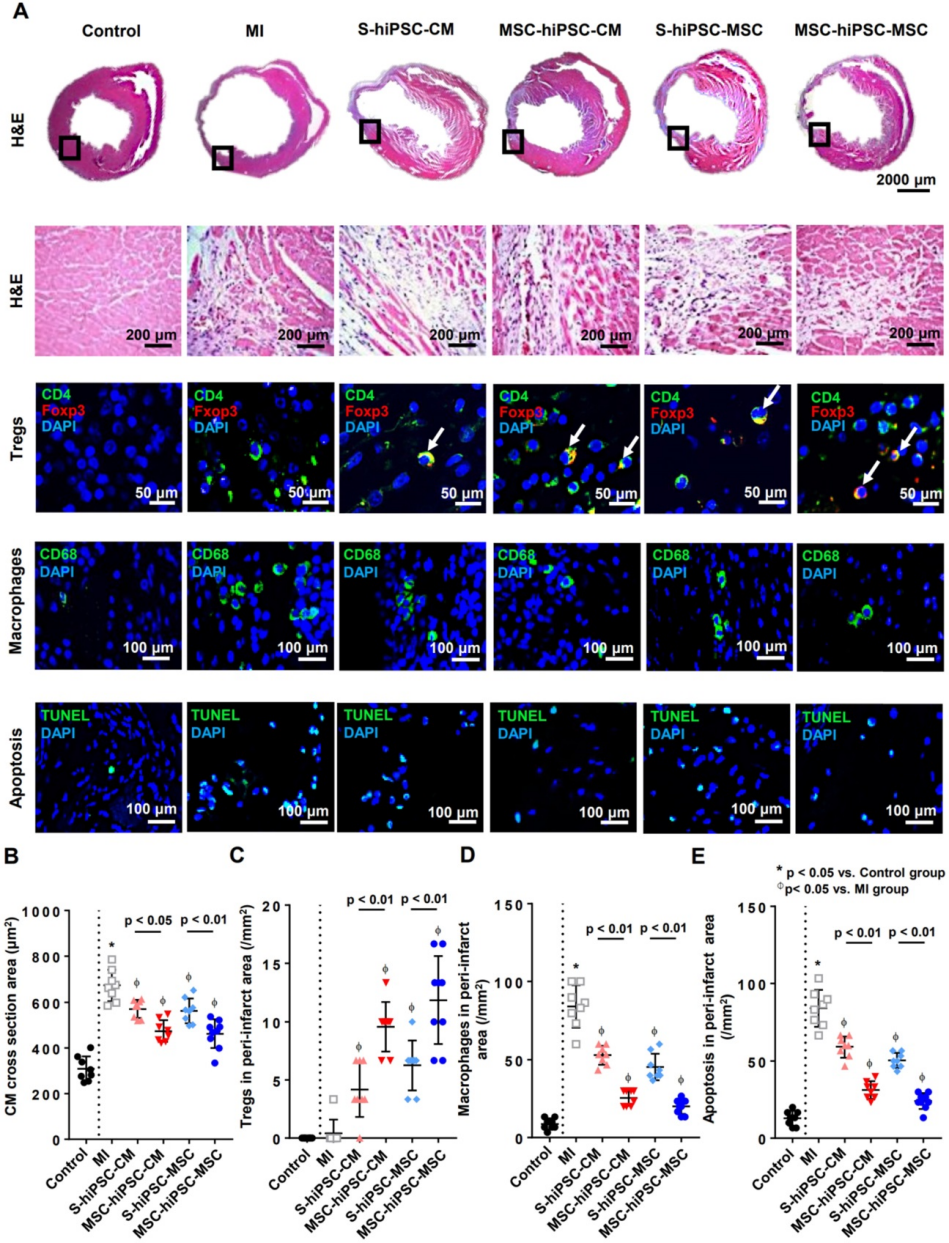
** Intravenous pre-transplantation systemic administration of hiPSC-MSCs increased Tregs and decreased the infiltration of macrophages and apoptosis in the peri-infarct area.** Hematoxylin and eosin (H&E) staining was performed to show the peri-infarct regions of the LV and to measure the cardiomyocyte cross sectional area in the peri-infarct area. To measure intramyocardial inflammation in the peri-infarct area, immunofluorescent staining of Tregs, macrophages and apoptotic cells in the peri-infarct area was performed 4 weeks after MI induction. Tregs were represented by CD4 (green) and Foxp3 (red) positive cells and cell nuclei were counterstained by DAPI (blue). Macrophages were represented by CD68 positive cells (green) and cell nuclei were counterstained by DAPI (blue). Apoptotic cells were represented by TdT-mediated dUTP Nick-End Labeling (TUNEL) positive cells (green) and cell nuclei were counterstained by DAPI (blue). Positive cells were counted under fluorescent microscopy and expressed as count per mm^2^. (**A**). Pre-transplantation systemic administration of hiPSC-MSCs significantly reduced the cross sectional area of native CMs in MSC-hiPSC-CM and MSC-hiPSC-MSC groups compared with S-hiPSC-CM and S-hiPSC-MSC groups, respectively (**B**). Pre-transplantation systemic administration of hiPSC-MSCs significantly increased myocardial Tregs (**C**) and decreased myocardial macrophages (**D**) and apoptotic cells (**E**) in MSC-hiPSC-CM and MSC-hiPSC-MSC groups compared with S-hiPSC-CM and S-hiPSC-MSC groups.

Hematoxylin and eosin (H&E) staining was performed to reveal the peri-infarct regions of the LV, and immunohistochemical staining with anti-mouse CD4, Foxp3, and CD68 to assess the number of CD4^+^ T cells, CD4/Foxp3^+^ Tregs and CD68^+^ macrophages, respectively after transplantation (**Figure 4A** and **Figure S5**). The phenotype of the macrophages was further characterized by immunostaining with anti-iNOS and anti-Arginase-1 for M1 and M2 patterns, respectively (**Figure S6**).

Histological assessment of a cross-section area of native mouse CMs was performed to assess CMs hypertrophy. Compared with the MI group, the CMs cross-sectional area was remarkably decreased in S-hiPSC-CM, MSC-hiPSC-CM, S-hiPSC-MSC and MSC-hiPSC-MSC groups (**Figure 4B**; *P*<0.05). Moreover, the CM cross-sectional area were further decreased in both MSC-hiPSC-CM and MSC-hiPSC-MSC groups at the peri-infarct regions of the LV after transplantation compared with the S-hiPSC-CM and S-hiPSC-MSC groups, respectively (**Figure 4B**; *P*<0.05).

The number of CD4/Foxp3^+^ Tregs was remarkably increased (**Figure 4C**) and the number of CD68^+^ macrophages was remarkably decreased (**Figure 4D**) in S-hiPSC-CM, MSC-hiPSC-CM, S-hiPSC-MSC and MSC-hiPSC-MSC groups compared with the MI group. Moreover, myocardial Tregs (**Figure 4C**; *P*<0.01) were further increased and myocardial macrophages (**Figure 4D**; *P*<0.01) were decreased in both MSC-hiPSC-CM and MSC-hiPSC-MSC groups at the peri-infarct regions of the LV after transplantation compared with the S-hiPSC-CM and S-hiPSC-MSC groups, respectively. There were no significant differences in the cellular infiltration of CD4^+^ T cells (**Figure S4**)*,* Tregs (**Figure 4C**) or macrophages (**Figure 4D**) at the myocardium after transplantation of hiPSC-CMs versus hiPSC-MSCs with or without systemic administration of hiPSC-MSCs. Interestingly, all macrophages detected at the peri-infarct regions exhibited an M2 phenotype (**Figure S6**).

TdT-mediated dUTP Nick-End Labeling (TUNEL) staining was performed to assess apoptosis at the peri-infarct regions of the LV after transplantation (**Figure 4A**). Compared with the control group, the number of apoptotic cells (12.9±1.7/mm^2^ versus 84.2±4.2/mm^2^; *P*<0.01) was markedly increased in the MI group (**Figure 4E**; *P*<0.01). Compared with the MI group, the number of apoptotic cells was significantly reduced in S-hiPSC-CM, MSC-hiPSC-CM, S-hiPSC-MSC and MSC-hiPSC-MSC groups (**Figure 4E**; *P*<0.05). The number of apoptotic cells were significantly decreased in both MSC-hiPSC-CMs and MSC-hiPSC-MSCs groups compared with the S-hiPSC-CM and S-hiPSC-MSC groups, respectively after transplantation (**Figure 4E**; *P*<0.01).

Our results showed that pre-transplantation systemic administration of hiPSC-MSCs regulated immune Tregs and macrophage infiltration and mitigation of apoptosis at the peri-infarct regions of the LV 4 weeks after intramyocardial transplantation of hiPSC-CMs or hiPSC-MSCs.

### Induction of neovascularization after transplantation

Immunohistochemical staining with alpha-smooth muscle antigen (α-SMA) and von Willebrand factor (vWF) was performed to assess neovascularization at the peri-infarct regions of the LV after cell transplantation (**Figure S7A-B**). Compared with the MI group, intramyocardial transplantation of hiPSC-CMs or hiPSC-MSCs with or without pre-transplantation systemic administration of hiPSC-MSCs significantly increased capillary density (**Figure S7C-D**; *P*<0.05). The capillary density was further significantly increased in both MSC-hiPSC-CM and MSC-hiPSC-MSC groups compared with the S-hiPSC-CM and S-hiPSC-MSC groups, respectively (**Figure S7C-D**; *P*<0.01). There were no differences between S-hiPSC-CM versus S-hiPSC-MSC groups or MSC-hiPSC-CM versus MSC-hiPSC-MSC groups, respectively (**Figure S7C-D**; *P*>0.05). Our results showed that pre-transplantation systemic administration of hiPSC-MSCs enhanced neovascularization at the peri-infarct regions of the LV 4 weeks after intramyocardial transplantation of hiPSC-CMs or hiPSC-MSCs.

### Immunomodulatory effect of systemic administration of hiPSC-MSCs

First, we defined cellular retention after systemic administration of hiPSC-MSCs in control mice without MI. Fluorescent imaging of the harvested splenocytes, livers, hearts, kidneys and lungs were performed to evaluate cellular engraftment of the labeled hiPSC-MSCs on day 1 and 7 after a single intravenous injection. As shown in **Figure S8A**, the majority of hiPSC-MSCs were distributed to the spleen, liver, heart, kidneys and lungs on day 1, but most had disappeared from the major organs, especially the heart and lungs, by day 7.

Then, we determined the optimal timing for the immunomodulatory effects of systemic hiPSC-MSC preconditioning prior to direct intramyocardial transplantation over a period of 11 days by measuring different populations of splenic immune cells. The splenic Tregs progressively increased after intravenous administration of hiPSC-MSCs from day 0 and reached a peak on day 7 (**Figure S8B**). There were no significant changes to the level of splenic CD4^+^, CD8^+^ or NK cells over time (**Figure S8C-E**). These findings further confirmed that the optimal immunomodulatory effects were achieved 7 days after systemic administration of hiPSC-MSCs.

**Figure 5:**
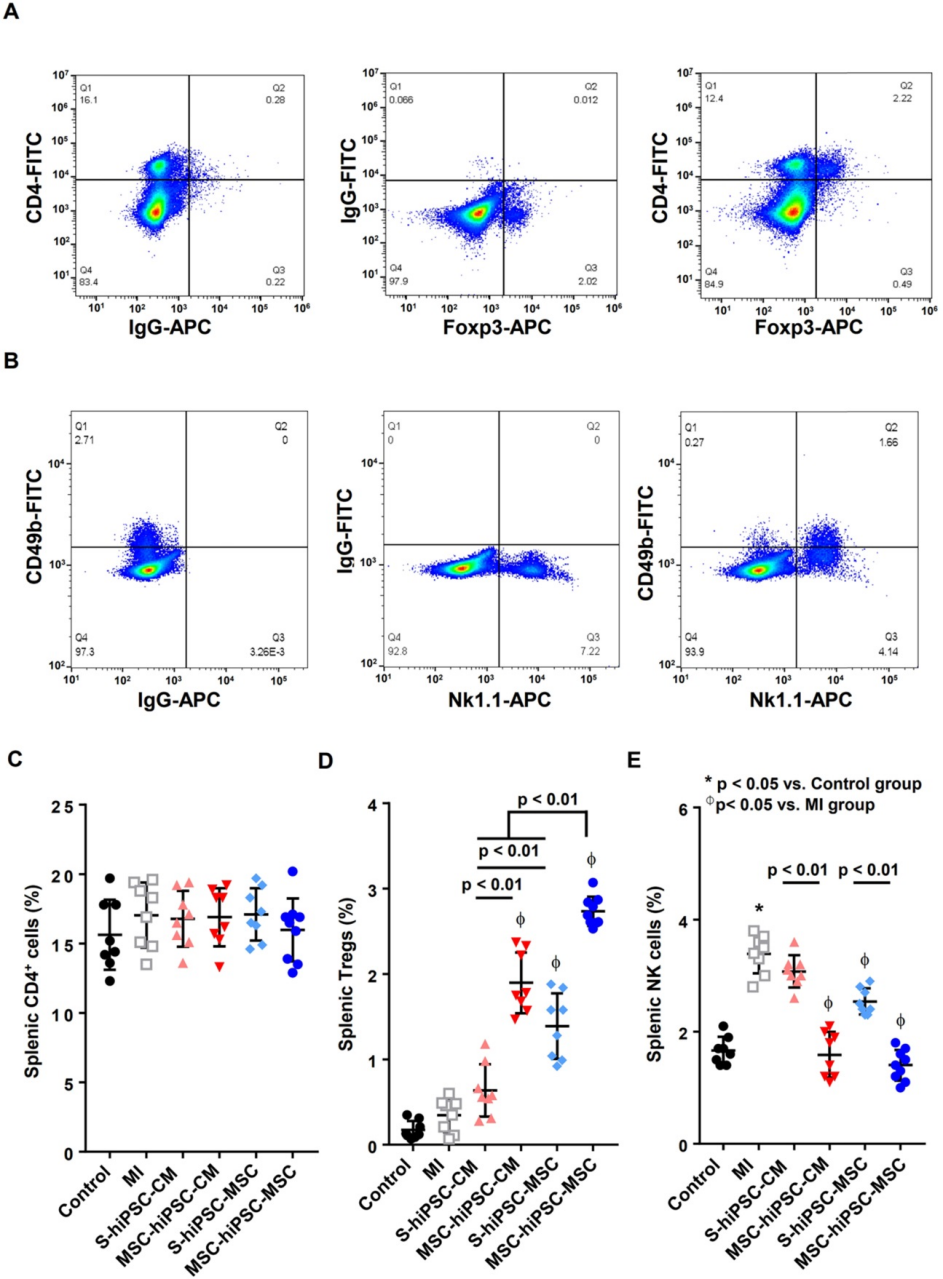
** Intravenous pre-transplantation systemic administration of hiPSC-MSCs increased Tregs and decreased NK cells in the spleen.** To identify splenic Tregs, splenocytes were stained with anti-mouse CD4-FITC and anti-mouse Foxp3-APC and analyzed by flow cytometry. “Fluorescence Minus One” (FMO) controls were obtained by omitting anti-mouse CD4-FITC antibody or anti-mouse Foxp3-APC antibody from the Tregs staining. Analysis of FMO controls for Tregs showed that omitting anti-mouse CD4-FITC antibody or anti-mouse Foxp3-APC antibody did not significantly change the frequency of positive cells for the other marker, confirming the validity of the gating strategy (**A**). To determine splenic NK cells, splenocytes were stained with anti-mouse CD49b-FITC and anti-mouse NK1.1-APC antibodies and analyzed by flow cytometry. FMO controls were obtained by omitting anti-mouse CD49b-FITC antibody or anti-mouse NK1.1-APC antibody from the NK cell staining. Analysis of FMO controls for NK cells showed that omitting anti-mouse CD49b-FITC antibody or anti-mouse NK1.1-APC antibody did not significantly change the frequency of positive cells for the other marker, confirming the validity of the gating strategy (**B**). CD4^+^ splenocytes (**C**), CD4^+^ Foxp3^+^ splenocytes (**D**) and CD49b^+^NK1.1^+^ splenocytes (**E**) were counted in all groups and expressed in percentage. Intravenous pre-transplantation systemic administration of hiPSC-MSCs increased splenic Tregs and decreased splenic NK cells.

Next, we evaluated the *in-vivo* effects of pre-transplantation systemic administration of hiPSC-MSCs on splenic CD4^+^ T cell, Tregs, and NK cell populations (**Figure 5**). The control of two-color staining of sham splenocytes is shown in **Figure 5A & B**. Compared with the MI group, the percentage of splenic CD4^+^ cells was not changed in S-hiPSC-CM, MSC-hiPSC-CM, S-hiPSC-MSC and MSC-hiPSC-MSC groups (**Figure 5C**; *P*>0.05). Compared with the MI group, intramyocardial transplantation of hiPSC-MSCs significantly increased splenic Tregs (**Figure 5D**; *P*<0.05); whereas intramyocardial transplantation of hiPSC-CMs had no such effect (**Figure 5D**; *P*>0.05). The splenic Tregs were significantly increased in both MSC-hiPSC-CM and MSC-hiPSC-MSC groups compared with S-hiPSC-CMs and S-hiPSC-MSCs, respectively (**Figure 5D**; *P*<0.01). Moreover, the percentage of splenic Tregs was higher in the S-hiPSC-MSC and MSC-hiPSC-MSC groups than the S-hiPSC-CM and MSC-hiPSC-CM groups (**Figure 5D**; *P*<0.01), respectively.

Compared with the control group, the percentage NK cell population was significantly increased in the MI group 4 weeks after induction of MI (**Figure 5E**; *P*<0.05). Compared with the MI group, S-hiPSC-MSC, as well as MSC-hiPSC-CM and MSC-hiPSC-MSC groups had a significantly reduced percentage of splenic NK cells (**Figure 5E**; *P*<0.05). The percentage splenic NK cell population were significantly decreased in both MSC-hiPSC-CM and MSC-hiPSC-MSC groups compared with the S-hiPSC-CM and S-hiPSC-MSC groups (**Figure 5E**; *P*<0.01).

Our results showed that pre-transplantation systemic administration of hiPSC-MSCs could provide immunomodulatory effects to enhance subsequent intramyocardial cell transplantation by inducing splenic activation of Tregs and suppressing activation of NK cells.

### Changes to cytokine profile in CD4^+^ splenocytes and hiPSC-MSCs co-cultured supernatant

**Figure 6:**
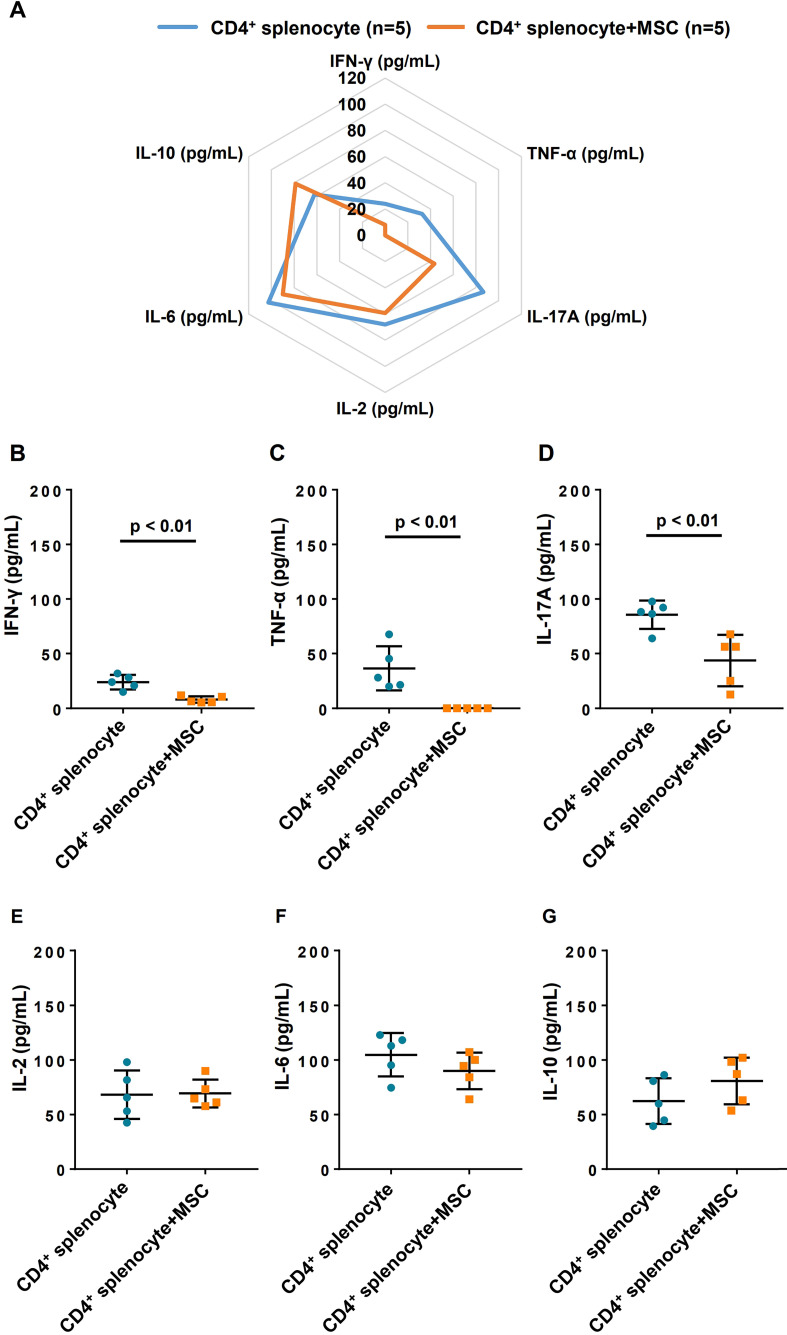
** Cytokine profiles changes after co-culture for 7 days with CD4 positive splenocytes and hiPSC-MSCs.** To demonstrate altered cytokine profiles, the supernatant level of cytokines was measured at day 7 (**A**). interferon (IFN)-γ **(B)** and tumor necrosis factor (TNF)-α (**C**), and interleukin (IL)-17A (**D**) in the supernatant reduced significantly after co-culture with hiPSC-MSCs for 7 days, compared with the cytokine level in the supernatant of the CD4 positive cell population alone. The cytokine level of IL-2 (**E**), IL-6 (**F**) and IL-10 (**G**) remained unchanged.

To investigate the immunomodulatory effect of hiPSC-MSCs on CD4^+^ cells, the changes to cytokine profile were measured in the supernatant after 7 days of co-culture with hiPSC-MSCs and CD4^+^ splenocytes (**Figure 6A**). After co-culturing of hiPSC-MSCs, the expression of tumor necrosis factor-α (**Figure 6B**) and interferon-γ (**Figure 6C**), which are related to T-helper cells (T_h_)1, as well as interleukin (IL)-17A (**Figure 6D**) related to T_h_17 by the CD4^+^ splenocytes were significantly decreased. On the contrary, T_h_2 related cytokines, including IL-2, IL-6 and IL-10 (**Figure 6E-G**) were unchanged. These data suggest that hiPSC-MSCs affected the release of T_h_1 and T_h_17-related cytokines in the CD4^+^ cell population and contributed to their immunomodulatory effects.

## Discussion

### Main findings

In this study, we provide novel evidence that pre-transplantation systemic intravenous administration of hiPSC-MSCs can induce immunomodulatory effects through activation of Tregs, suppression of NK cells, and modulation of the expression of cytokine profiling of CD4^+^ cells to enhance engraftment and survival of cells transplanted intramyocardially for treatment of MI. Increased survival of transplanted hiPSC-MSCs or hiPSC-CMs following systemic hiPSC-MSCs pre-conditioning was associated with further improvement in LV function, likely mediated by enhanced neovascularization and reduced cellular inflammation and apoptosis at the peri-infarct zone. Finally, there was no tumor formation at the injection site or other sites over the myocardium or other organs.

### Immunomodulatory effects of hiPSC-MSCs

Emerging evidence suggests that pluripotent stem cell-derived cell types such as hiPSC-MSCs 14 and hiPSC-CMs 20, 21 are promising allogenic “*off-the-shelf*” cell sources for cardiac repair after MI. Our recent study showed that intramyocardial transplantation of hiPSC-MSCs improved neovascularization and LV function in a porcine model of post-MI HF 14. Nevertheless, their poor survival and engraftment due to immune rejection and inflammation despite immunosuppression therapies (steroid and cyclosporine) remain major hurdles to their therapeutic application. Despite the potential immune-privilege of hiPSC-MSCs due to their limited expression of HLA at baseline, upregulation of HLA on hiPSC-MSCs can be triggered by exposure to interferon-γ 15. Moreover, local delivery of hiPSC-MSCs into the myocardium may have limited immunomodulatory effects. In this study, we observed only a modest increase in the percentage of splenic Tregs after intramyocardial hiPSC-MSCs transplantation compared with hiPSC-CMs transplantation. It remains unclear whether the immunomodulatory effects of hiPSC-MSCs can be further enhanced by pre-transplantation systemic administration of MSCs.

Previous animal studies have demonstrated that pre-transplantation systemic administration of MSCs 4-7 days rather than 0-3 days before heart transplantation can prolong graft survival 22-26. The improved engraftment has been attributed to increased circulating Tregs following systemic administration of MSCs 27. In this study, we aimed to investigate the immunomodulatory effects of hiPSC-MSCs and thus immunosuppressive agents were not administrated before systemic or intramyocardial cellular transplantation. Indeed, splenic Tregs increased to a peak level 7 days following systemic intravenous administration of hiPSC-MSCs in normal mice without MI. Next, our results demonstrated that pre-transplantation systemic administration of hiPSC-MSCs in the MSC-hiPSC-MSC group further significantly increased splenic Tregs and decreased splenic NK cells compared with intramyocardial hiPSC-MSCs transplantation alone.

Our *in-vitro* co-culture study demonstrated that hiPSC-MSCs could modify the cytokine expression profile of splenic CD4^+^ T cells with a decrease in interferon-γ that mediates the cellular immune response, as well as pro-inflammatory cytokines tumor necrosis factor-α, and IL-17A. Most importantly, these immunomodulatory effects were associated with improved survival of subsequent intramyocardially transplanted hiPSC-MSCs or hiPSC-CMs. On the other hand, we observed no significant changes to the expression of Th2-related cytokines (IL-2, IL-6 and IL-10) by splenic T cells after co-culture with hiPSC-MSCs. This is likely because MSCs have direct effects on Th1-related and Th17-related cytokine expression by T cells, but inhibition of Th-2 related cytokines is mediated indirectly via induction of Tregs and could be determined by this *in-vitro* co-culture experiment 28.

In this study, we also determined whether the immunomodulatory effects of systemic administration of hiPSC-MSCs could induce tolerance of only one cell type, i.e., hiPSC-MSCs or be extended to other cell types, such as isogenic hiPSC-CMs derived from the same iPSC line. Our results showed that intramyocardial engraftment and survival of hiPSC-CMs and hiPSC-MSCs were significantly increased after pre-transplantation systemic administration of hiPSC-MSCs. Moreover, Tregs were also significantly increased and macrophage infiltration at the myocardium was decreased after transplantation of either hiPSC-CMs or hiPSC-MSCs compared with those without systemic administration of hiPSC-MSCs. Interestingly, we detected only macrophages with an M2 phenotype at the peri-infarct LV regions that decreased after either hiPSC-CMs or hiPSC-MSCs transplantation and reduced further after pre-transplantation of hiPSC-MSCs. Prior studies have demonstrated that macrophages begin to adopt a reparative M2 phenotype in the later phase of MI 29. The decreased number of M2 macrophages after cellular transplantation is likely related to reduced local CM injury after either hiPSC-CMs or hiPSC-MSCs transplantation.

Nevertheless, we observed greater improvements in engraftment and survival for hiPSC-MSCs than hiPSC-CMs, likely due to the local immunomodulatory effects of hiPSC-MSCs as Tregs and macrophage infiltration were insignificantly higher and lower respectively in mice that underwent intramyocardial transplantation of hiPSC-MSCs versus hiPSC-CMs. These findings are consistent with our recent observation of the superior engraftment and survival of hiPSC-MSCs compared with human embryonic stem cell-derived CMs, attributed to local immunomodulatory effects of hiPSC-MSCs 14. Nevertheless, these results also highlight the relatively weaker immunomodulatory effects of locally administered hiPSC-MSCs. Whether a combination of pre-treatment systemic administration with local intramyocardial injection of hiPSC-MSCs can further improve the engraftment and survival of hiPSC-CMs deserves future investigation.

### Mechanism of cell engraftment and cardiac function improvement

Our results showed that LV function significantly improved following intramyocardial transplantation of hiPSC-MSCs or hiPSC-CMs with pre-transplantation systemic intravenous administration of hiPSC-MSCs. Despite the significant improvement in cell survival, size of MI was not affected. Although systemic hiPSC-MSCs pre-conditioning increased the survival and engraftment of hiPSC-MSCs more than that of hiPSC-CMs, a similar increase in neovascularization and decreased myocardial inflammation and apoptosis at the peri-infarct site were observed in both groups. It is possible that some of these beneficial effects were due to systemic administration of hiPSC-MSCs even though hiPSC-MSCs were rarely detected 7 days after intravenous injection. More importantly, those changes were associated with significant improvement in LV function after MI. We observed no human CMs after intramyocardial transplantation of hiPSC-MSCs with or without pre-transplantation systemic intravenous administration of hiPSC-MSCs, indicating the lack of any trans-differentiation of hiPSC-MSCs to human CMs. Taken together, our results are consistent with recent observations that the major mechanisms of action of cellular transplantation are mediated via their paracrine effects 30, 31 and anti-inflammatory effects 32, rather than direct myocardial remuscularization. Our results support these findings and further demonstrate that increased transplanted cell survival by pre-transplantation systemic intravenous administration of hiPSC-MSCs can further enhance these paracrine effects of intramyocardial transplantation of hiPSC-MSCs or hiPSC-CMs.

In conclusion, the results of this study provide proof-of-principle data to support the potential therapeutic application of pre-transplantation systemic administration of hiPSC-MSCs modulated systemic Tregs and NK cells and improve the survival of intramyocardial transplantation of different cell sources, including hiPSC-MSCs and hiPSC-CMs for MI. Future studies should be performed to determine whether these immunomodulatory effects of systemic hiPSC-MSCs can be further enhanced by co-administration of immunosuppressive agents or used to improve the engraftment of bioengineered cellular patches.

This study has limitations. First, single intravenous administration of hiPSC-MCSs was given 1 week before the induction of MI as coronary artery ligation and intramyocardial cellular transplantation were performed in the same setting due to the limitation of this small animal modal of MI in this study. This approach cannot mimic the clinical scenario of patients presented with MI. Nonetheless, our results provide important proof-of principle data to support future studies on the immunomodulation effects of systemic administration of hiPSC-MSCs in large animal models of ischemic HF 14. Second, we tested only a single intravenous infusion of 5x10^5^ hiPSC-MSCs 7 days before MI induction and intramyocardial cellular transplantation. Moreover, the optimal approach, including dosage and number of pre-transplantation injections of hiPSC-MSCs remains unclear. Third, direct intramyocardial cellular transplantation was performed immediately after induction of MI. The highly inflammatory environment of the myocardium may have confounded the beneficial effects of hiPSC-MSCs. Our recent studies have demonstrated the potential therapeutic and immunomodulatory effects of local intramyocardial transplantation of hiPSC-MSCs in a porcine model of chronic HF after MI 14. Nevertheless, it remains unclear whether the immunomodulatory effects of systemic administration of hiPSC-MSCs can be observed in chronic post-MI HF. Forth, although we detected significant changes to the cytokine expression of CD4^+^ splenocytes with hiPSC-MSCs co-culture *in-vitro*, changes to the systemic and local cytokine profiles were not measured. In addition to changes in the systemic and local infiltration of Tregs and NK cells, the altered expression of different anti-inflammatory and anti-apoptotic cytokines should also contribute to the immunomodulatory effect of hPSC-MSCs 33. Further *in-vivo* studies are required to clarify the potential roles of different cytokine expressions related to the immunomodulatory effects of hiPSC-MSCs. Final, the current approach to characterize the M1/M2 polarization paradigm of cardiac macrophage in this study was mainly based on the presence of the surface markers rather than actual expression of those proteins, such as Arginase-1 34.

## Supplementary Material

Supplementary methods and figures.

## Abbreviations

α-SMA: alpha-smooth muscle antigen
BM: bone marrow
CM: cardiomyocyte
+dP/dt_max_: maximal positive pressure derivative
ESPVR: end systolic pressure-volume relationship
FS: fractional shortening
HF: heart failure
hiPSC: human induced pluripotent stem cell
H&E: hematoxylin and eosin
HLA: human leukocyte antigen
IL: interleukin
LV: left ventricle
LVEF: left ventricular ejection fraction
LVESD: LV end-systolic dimension
LVEDD: LV end-diastolic dimension
MI: myocardial infarction
MSC: mesenchymal stromal cell
NK: natural killer
PCR: polymerase chain reaction
T_h_: T-helper cell
Tregs: regulatory T cells
TUNEL: TdT-mediated dUTP Nick-End Labeling
vWF: von Willebrand factor

## References

[1] Benjamin EJ, Muntner P, Alonso A, Bittencourt MS, Callaway CW, Carson AP (2019). Heart Disease and Stroke Statistics-2019 Update: A Report From the American Heart Association. Circulation.

[2] Drazner MH (2019). Angiotensin receptor-neprilysin inhibition (arni) therapy and reverse remodeling in heart failure with reduced ejection fraction. JAMA.

[3] Bolli R, Ghafghazi S (2017). Stem cells: Cell therapy for cardiac repair: what is needed to move forward?. Nat Rev Cardiol.

[4] Assmus B, Alakmeh S, De Rosa S, Bönig H, Hermann E, Levy WC (2016). Improved outcome with repeated intracoronary injection of bone marrow-derived cells within a registry: rationale for the randomized outcome trial REPEAT. Eur Heart J.

[5] Traverse JH, Henry TD, Ellis SG, Pepine CJ, Willerson JT, Zhao DX (2011). Effect of intracoronary delivery of autologous bone marrow mononuclear cells 2 to 3 weeks following acute myocardial infarction on left ventricular function: the LateTIME randomized trial. JAMA.

[6] Traverse JH, Henry TD, Pepine CJ, Willerson JT, Zhao DX, Ellis SG (2012). Effect of the use and timing of bone marrow mononuclear cell delivery on left ventricular function after acute myocardial infarction: the TIME randomized trial. JAMA.

[7] Kim SW, Houge M, Brown M, Davis ME, Yoon YS (2014). Cultured human bone marrow-derived CD31(+) cells are effective for cardiac and vascular repair through enhanced angiogenic, adhesion, and anti-inflammatory effects. J Am Coll Cardiol.

[8] Wang YL, Zhang G, Wang HJ, Tan YZ, Wang XY (2018). Preinduction with bone morphogenetic protein-2 enhances cardiomyogenic differentiation of c-kit+ mesenchymal stem cells and repair of infarcted myocardium. Int J Cardiol.

[9] Tongers J, Losordo DW, Landmesser U (2011). Stem and progenitor cell-based therapy in ischaemic heart disease: promise, uncertainties, and challenges. Eur Heart J.

[10] Kretlow JD, Jin Y-Q, Liu W, Zhang WJ, Hong T-H, Zhou G (2008). Donor age and cell passage affects differentiation potential of murine bone marrow-derived stem cells. BMC Cell Biol.

[11] Wagner W, Bork S, Horn P, Krunic D, Walenda T, Diehlmann A (2009). Aging and replicative senescence have related effects on human stem and progenitor cells. PLoS One.

[12] Lian Q, Zhang Y, Zhang J, Zhang HK, Wu X, Zhang Y (2010). Functional mesenchymal stem cells derived from human induced pluripotent stem cells attenuate limb ischemia in mice. Circulation.

[13] Lian Q, Zhang Y, Liang X, Gao F, Tse HF (2016). Directed Differentiation of Human-Induced Pluripotent Stem Cells to Mesenchymal Stem Cells. Methods Mol Biol.

[14] Liao S, Zhang Y, Ting S, Zhen Z, Luo F, Zhu Z (2019). Potent immunomodulation and angiogenic effects of mesenchymal stem cells versus cardiomyocytes derived from pluripotent stem cells for treatment of heart failure. Stem Cell Res Ther.

[15] Sun YQ, Zhang Y, Li X, Deng MX, Gao WX, Yao Y (2015). Insensitivity of human iPS cells-derived mesenchymal stem cells to interferon-γ-induced HLA expression potentiates repair efficiency of hind limb ischemia in immune humanized NOD SCID Gamma mice. Stem Cells.

[16] Fu QL, Chow YY, Sun SJ, Zeng QX, Li HB, Shi JB (2012). Mesenchymal stem cells derived from human induced pluripotent stem cells modulate T-cell phenotypes in allergic rhinitis. Allergy.

[17] Gao F, Chiu S, Fu Q, Motan DAL, Zhang Z, Chen L (2016). Mesenchymal stem cells and immunomodulation: Current status and future prospects. Cell Death Dis.

[18] Zhao L, Chen S, Yang P, Cao H, Li L (2019). The role of mesenchymal stem cells in hematopoietic stem cell transplantation: prevention and treatment of graft-versus-host disease. Stem Cell Res Ther.

[19] Reinders MEJ, van Kooten C, Rabelink TJ, de Fijter JW (2018). Mesenchymal stromal cell therapy for solid organ transplantation. Transplantation.

[20] Yuji S, Toshihito G, Tatsuichiro S, Yuko W, Hajime I, Yuki T (2016). Allogeneic transplantation of iPS cell-derived cardiomyocytes regenerates primate hearts. Nature.

[21] Hamad S, Derichsweiler D, Papadopoulos S, Nguemo F, Šarić T, Sachinidis A (2019). Generation of human induced pluripotent stem cell-derived cardiomyocytes in 2D monolayer and scalable 3D suspension bioreactor cultures with reduced batch-to-batch variations. Theranostics.

[22] Casiraghi F, Perico N, Cortinovis M, Remuzzi G (2016). Mesenchymal stromal cells in renal transplantation: opportunities and challenges. Nat Rev Nephrol.

[23] Zhou HP, Yi DH, Yu SQ, Sun GC, Cui Q, Zhu HL (2006). Administration of donor-derived mesenchymal stem cells can prolong the survival of rat cardiac allograft. Transplant Proc.

[24] Chabannes D, Hill M, Merieau E, Rossignol J, Brion Rg, Soulillou JP (2007). A role for heme oxygenase-1 in the immunosuppressive effect of adult rat and human mesenchymal stem cells. Blood.

[25] Eggenhofer E, Popp FC, Mendicino M, Silber P, Van' T Hof W, Renner P (2013). Heart grafts tolerized through third-party multipotent adult progenitor cells can be retransplanted to secondary hosts with no immunosuppression. Stem Cells Transl Med.

[26] Obermajer N, Popp FC, Soeder Y, Haarer J, Geissler EK, Schlitt HJ (2014). Conversion of Th17 into IL-17A(neg) regulatory T cells: a novel mechanism in prolonged allograft survival promoted by mesenchymal stem cell-supported minimized immunosuppressive therapy. J Immunol.

[27] Popp FC, Eggenhofer E, Renner P, Slowik P, Lang SA, Kaspar H (2008). Mesenchymal stem cells can induce long-term acceptance of solid organ allografts in synergy with low-dose mycophenolate. Transpl Immunol.

[28] Duffy MM, Ritter T, Ceredig R, Griffin MD (2011). Mesenchymal stem cell effects on T-cell effector pathways. Effector pathways. Stem Cell Res Ther.

[29] O'Rourke SA, Dunne A, Monaghan MG (2019). The role of macrophages in the infarcted myocardium: Orchestrators of ECM remodeling. Front Cardiovasc Med.

[30] Ranganath SH, Levy O, Inamdar MS, Karp JM (2012). Harnessing the mesenchymal stem cell secretome for the treatment of cardiovascular disease. Cell Stem Cell.

[31] Gao L, Mei S, Zhang S, Qin Q, Li H, Liao Y (2020). Cardio-renal Exosomes in Myocardial Infarction Serum Regulate Proangiogenic Paracrine Signaling in Adipose Mesenchymal Stem Cells. Theranostics.

[32] Luger D, Lipinski MJ, Westman PC, Glover DK, Dimastromatteo J, Frias JC (2017). Systemic anti-inflammatory effects improve left ventricular dysfunction in acute myocardial infarction and ischemic cardiomyopathy. Circ Res.

[33] Lee RH, Pulin AA, Seo MJ, Kota DJ, Ylostalo J, Larson BL (2009). Intravenous hMSCs improve myocardial infarction in mice because cells embolized in lung are activated to secrete the anti-inflammatory protein TSG-6. Cell Stem Cell.

[34] Nahrendorf M, Swirski FK (2016). Abandoning M1/M2 for a Network Model of Macrophage Function. Circ Res.

