# Supplementary material for: Immunomodulation by systemic administration of human-induced pluripotent stem cell-derived mesenchymal stromal cells to enhance the therapeutic efficacy of cell-based therapy for treatment of myocardial infarction
Source: Theranostics. 2021 Jan 1;11(4):1641–54. doi: 10.7150/thno.46119 (PMC7778603; doi:10.7150/thno.46119)
Supplement: Supplementary file 1 — Supplementary methods and figures. [file thnov11p1641s1.pdf]

## Supplemental materials

## Supplemental methods

All procedures involving animals were approved by the Committee on the Use of Live Animals in Teaching and Research at the University of Hong Kong, Hong Kong (Project number 3999-16).

**Human induced pluripotent stem cells culture, differentiation and preparation**

In this study, human induced pluripotent stem cells (hiPSC) line IMR90-iPSCs (WiCell Research Institute, Madison, WI, USA) was used and differentiated into hiPSC-derived mesenchymal stromal cells (hiPSC-MSCs) as described previously [1-4]. Briefly, hiPSC-MSCs were purified by sorting for $CD105^{+}/CD24^{-}$ cells using the MoFlo Cell sorting system (Beckman-coulter, USA). Sorted cells were cultured and expanded in MSC medium (Stemcells Technologies, Toronto, Canada). The hiPSC-MSCs were characterized by the expression of CD44, CD49a and e, CD73, CD105, and CD166; and absence of CD45, CD34, and CD133 expression [1-4]. Furthermore, hiPSC-MSCs are able to differentiate into osteoblasts, adipocytes, and chondroblasts. Passage 4-6 hiPSC-MSCs were used for transplantation. For intravenous administration, $5x10^5$ hiPSC-MSCs were prepared in 100 $\mu$L of normal saline and injected into the tail vein.

For hiPSC-derived cardiomyocytes (hiPSC-CMs) generation, IMR90-iPSCs were differentiated using a small molecule-directed differentiation protocol as per our previous studies [5, 6]. Beating clusters of hiPSC-CMs were observed 21-24 days after differentiation and cultured for a further 30 days to reach maturity. A suspension of hiPSC-CMs was obtained by enzymatic dissociation using 2 $\mu$g/ml collagenase IV (Life Technologies, US).

For cell tracking after transplantation, one million hiPSC-MSCs or hiPSC-CMs were stained with 5 $\mu$g/mL DiR cell-labeling solution (Life Technologies Invitrogen, US) for 15 min at 37 °C, washed and re-suspended in 30 $\mu$l of fresh plain medium for intramyocardial transplantation [7].

**Animal model and transplantation**

In this study, ICR mice were used since their stronger pro-inflammatory response would more easily demonstrate the anti-inflammatory effects of transplanted cells [8]. All animals received an intravenous injection of either 100 $\mu$l of saline or 5x10$^5$ hiPSC-MSCs prepared in 100 $\mu$L of normal saline via the tail vein 1 week before induction of myocardial infarction (MI), achieved by direct ligation of the left anterior descending coronary artery as previously described [9]. Prior studies have shown that pre-transplantation systemic administration of 5x10$^5$ MSCs can prolong graft survival (heart or kidney) in a mouse model [10, 11]. Our pilot data also demonstrated that this dosing of 5x10$^5$ hiPSC-MSCs administrated intravenously alone one week before induction of MI had no significant therapeutic effect on left ventricular (LV) function, and thus avoided any confounding effects when assessing therapeutic efficacy of direct intramyocardial cellular transplantation (***Figure S9A***). Moreover, our anti-human CD105 immunostaining results showed that no intravenous transplanted hiPSC-MSCs were detected in the infarcted heart (***Figure S9B***). 10 min after induction of MI, animals were randomized to receive direct intramyocardial injection of culture medium (30 $\mu$l), or 1x10$^6$ hiPSC-MSCs or hiPSC-CMs at three different LV sites near the infarct border zone.

As shown in ***Figure 1***, six groups of adult male ICR mice aged 12–16 weeks were used: (1) intravenous administration of saline alone (*Control group*); (2) intravenous administration of saline before MI and intramyocardial injection of culture medium (*MI group*); (3) intravenous administration of saline before MI and intramyocardial transplantation of 1x10$^6$ hiPSC-CMs (*S-hiPSC-CM group*); (4) intravenous administration of 5x10$^5$ hiPSC-MSCs before MI and intramyocardial transplantation of 1x10$^6$ hiPSC-CMs (*MSC-hiPSC-CM group*); (5) intravenous administration of saline before MI and intramyocardial transplantation of 1x10$^6$ hiPSC-MSCs (*S-hiPSC-MSC group*); and (6) intravenous administration of 5x10$^5$ hiPSC-MSCs before MI and intramyocardial transplantation of 1x10$^6$ hiPSC-MSCs (*MSC-hiPSC-MSC group*). Three mice in each group were sacrificed immediately and on day 7 for fluorescent imaging analysis of DiR signal. The remaining mice from six groups (control group (n=8), MI group(n=8), S-hiPSC-CM group (n=8), S-hiPSC-MSC group (n=8), MSC-hiPSC-CM group (n=8), MSC-hiPSC-MSC group (n=9)) were sacrificed on day

28.

On day 28 after intramyocardial transplantation, cardiac function was assessed by transthoracic echocardiogram (Vivid-I, GE Medical Systems, Milwaukee, WI, USA) and invasive hemodynamic pressure-volume loop measurements (SciSence Techology Inc., Canada) [7,9].

**Echocardiographic examination and invasive hemodynamic assessment**

Serial echocardiography was performed to assess left ventricular (LV) function at 28 days after intramyocardial transplantation using a high-resolution ultrasound system (Vivid-I, GE Medical Systems, Milwaukee, WI) and a 10S-RS sector transducer (4.4–11.5 MHz). All echocardiographic examinations were performed by an experienced operator blinded to the treatment received by each animal. In brief, mice were anesthetized with an intraperitoneal injection of ketamine (100 mg/kg) and xylazine (20 mg/kg) and examined in the left lateral decubitus position with an ultrasound gel pad positioned on the anterior chest wall. Standard M-mode parameters, including LV end-systolic dimension (LVESD), and LV end-diastolic dimension (LVEDD) were measured to calculate LV fractional shortening (FS) and LV ejection fraction (LVEF) according to the American Society of Echocardiography recommendations [7]. The mean value of three cardiac cycles was used for each individual mouse at each time point.

Four weeks after intramyocardial cell transplantation, animals were anesthetized and mechanically ventilated as described as before [7]. A 1.2-Fr pressure-volume (PV) conductance catheter connected to an advantage PV Loop system (Science Inc., Ontario, Canada) was inserted into the LV cavity via the right carotid artery. Hemodynamic parameters including maximal and negative pressure derivative (±dP/dt) were recorded using LabScribe Recording and Analysis Software (Scisence Inc., Ontario, Canada). The slope of the end systolic pressure-volume relationship (ESPVR) was measured before and after inferior vena cava occlusion to produce baseline and dynamic PV loops under changes in preload [9]. All PV loop parameters were measured in a blinded fashion.

**Tissue collection and histological assessment**

To quantify and detect the engraftment of transplanted cells, animal hearts were harvested after sacrifice for epi-fluorescent imaging using an IVIS® Spectrum optical imaging system under field of view (FOV) 3.9 x 3.9 cm (PerkinElmer, Inc. MA, USA) [12] The engrafted DiR labeled cells were visualized under a 750 nm/800 nm excitation/emission wavelength with fluorescent intensity represented by radiant efficiency. To estimate the survival rate of the transplanted cells after intramyocardial injection, the averaged radiant efficiency in each group at day 0, 7 or 28 was calculated. The survival rate in each group at day 7 or 28 was expressed as the rate of the mean of radiant efficiency at day 7 or 28 and the mean of radiant efficiency at day 0.

Next, the harvested hearts were immediately fixed in ice-cold 4% buffered formalin, followed by gradient alcohol dehydration, then embedded in paraffin for sectioning into 5 μm slices prior to histological assessment. Paraffin sections of heart tissue were used to determine infarct size using Trichrome Masson's stain. Infarct size was represented by the ratio of infarct area (characterized by fibrotic tissue strained blue) to LV area (characterized by myocardial tissue stained red and fibrotic tissues) and measured using AxioVision Rel. 4.5 software (Zeiss, GmbH, Oberkochen, Germany) at three cross sections. The mean value of the three sections was calculated to represent infarct size. The cross-sectional area of native cardiomyocytes in the peri-infarct area was measured in 15 cardiomyocytes from three different hematoxylin and eosin (H&E) sections using AxioVision Rel. 4.5 software (Zeiss, GmbH, Oberkochen, Germany). The mean value of these 15 cells was calculated to represent the cross-sectional area. Animals with no evidence of MI (determined by Masson Trichrome staining) were excluded from the analysis (n=2).

Immunohistochemical staining was performed to assess cell engraftment, neovascularization, myocardial infiltration of inflammatory cells and apoptosis. Anti-human CD105 antibody (1:100, LS-B2165, LifeSpan BioSciences, USA) and anti-human Troponin I antibody (1:100, ab52862, Abcam, USA) staining was used to evaluate engraftment of the transplanted hiPSC-MSCs and hiPSC-CMs, respectively. The number of cells that stained positive was counted over the peri-infarct regions in

each treatment group and expressed as count per $mm^2$. Staining with anti-mouse alpha-smooth muscle antigen (α-SMA) (1:200, M0851,Sigma-Aldrich, St Louis, MO, USA) and anti-mouse von Willebrand factor (vWF) (1:200, ab7356, Merck Millipore, USA) was performed to assess neovascularization at the peri-infarct regions after transplantation [13]. Vessel density was determined as absolute number of staining positive microvessels over the peri-infarct regions in each treatment group and expressed as count per $mm^2$. Heart sections were also performed immunostaining to estimate the number of leukocytes in the peri-infarct area. CD4/Foxp3$^+$ Tregs infiltration was evaluated by antibody staining with anti-mouse CD4 (1:100, ab183685, Abcam, USA) with and without anti-mouse Foxp3 (1:100, eBio7979, eBioscience, USA) [9]. Macrophage infiltration was assessed by antibody staining with anti-mouse CD68 (1:200, ab31630, Abcam, USA) independent of their polarization [14]. The polarization of macrophages was further characterized by immunostaining with anti-iNOS (1:100, ab15323, Abcam, USA) and anti-Arginase 1 (1:100, ab60176, Abcam, USA) for M1 and M2 phenotypes, respectively [15]. The number of apoptotic cells in the peri-infarct area was assessed using a TdT-mediated dUTP Nick-End Labeling (TUNEL) kit (11684809910, Roche Diagnostics, Indianapolis, IN, USA). The positive and negative controls of TUNEL staining are shown in ***Figure S10***. All data were measured in six random 40X fields from three different cross sections in a blinded fashion. Images of all sections were captured and analyzed by AxioVision Rel. 4.5 software (Zeiss, GmbH, Oberkochen, Germany).

**Flow cytometry analysis**

Splenic T cells and natural killer (NK) cells were analyzed by flow cytometry (Beckman-coulter, USA). Under anesthetic, the spleen was harvested from all animals. A single cell suspension of splenic lymphocytes was prepared by meshing through 100 $\mu$m nylon cell strainers (Falcon, NY, USA) and red blood cells removed by ACK lysing solution (Invitrogen, USA). One million splenocytes were then blocked by the purified anti-mouse CD16/32 Antibody (1:100 dilution, Biolegend, San Diego, CA) and 7AAD (1:50, BD Bioscience, USA) in flow buffer containing PBS, 1% BSA, 2 mM EDTA, 0.01% $NaN_3$ for 10 min at 4 ˚C. After washing, these cells were subsequently used for staining of mouse CD4, CD8, Foxp3 (Mitenyl Biotech, GmBH), and NK1.1 and CD49b respectively (Biolegend, San Diego, CA). For Treg staining,

one million splenocytes were stained with 1:20 anti-mouse CD4-FITC for 15 min and then fixed and made permeable with fixation-permeabilization buffers (Mitenyl Biotech, GmBH) for 30 min on ice. Intracellular staining of Foxp3 was done by incubating with 1:20 anti-mouse Foxp3-APC antibody. (Mitenyl Biotech, GmBH). NK cells were stained using 1:100 anti-mouse NK1.1-APC and anti-mouse CD49b-FITC antibodies for 30 min on ice (Biolegend, San Diego, CA). Cytotoxic T cells were stained using 1:20 ant-mouse CD8-APC antibody for 15 min on ice. Stained cells were analyzed using a Cytomic FC500 flow cytometer (Beckman Coulter, Miami, FL). Fluorescence minus one (FMO) controls, obtained by omitting a single antibody from the labeling antibody cocktail, were used for multicolor flow cytometry panels to determine the gates to be set [16]. The population of splenic T cells, Tregs and NK cells detected by flow cytometry was calculated from the percentage of cells in the number of viable cells. The number of Tregs was expressed as Foxp3$^+$ cells in a proportion of pre-gated CD4$^+$ cells. Flow images were analyzed by FlowJo software (V10.7.1, Becton, USA).

**Polymerase chain reaction (PCR) analysis**

PCR of human cell DNA was performed to confirm cell retention after transplantation [17]. Genomic DNA was obtained from formalin-fixed paraffin-embedded tissues using a QIAamp DNA FFPE Tissue Kit according to the protocol supplied (56404, QIAGEN, USA). Formalin-fixed paraffin-embedded tissues were sectioned into 10 $\mu$m slices. Eight 10 $\mu$m thick slices were deparaffinized by xylene and then washed with absolute ethanol. Next, tissues were digested by proteinase K. Then, genomic DNA was extracted using DNA extraction solution [18]. After genomic DNA extraction, PCR was performed with DNA polymerase (TAK R010A, Takara Bio, WI, USA). Human DNA was detected using human GAPDH promoter and mouse DNA by mouse mitochondria promoter. Primers homo GAPDH-F: AATCCCATCACCATCTTCCAG; and homo GAPDH-R: AAGTGAGCCCCAGCCTTC were used to amplify a genomic DNA fragment in the human GAPDH promoter. Primers Mus mitochondrion-F: AGCATGATACTGACATTTTGTAGACGT and Mus mitochondrion-R: GATAACAGTGTACAGGTTAATTACTCTCTTCTG were used to amplify a genomic DNA fragment in the mouse mitochondria promoter. PCR protocol was 30 cycles at 98 °C for 10 s, 55 °C for 15 s, and 72 °C for 30 s. The amplified

products were determined by electrophoresis in 1.5% agarose gel containing ethidium bromide. The results were expressed as the ratio of the density of human GAPDH products to density of mouse mitochondria products. The density of PCR products was analyzed by ENDURO™ GDS Gel Documentation System (Labnet International Inc., Edison, USA)

**Immunomodulatory effects of hiPSC-MSCs**

First, the cellular distribution and retention of hiPSC-MSCs in major organs after a single intravenous injection were evaluated in an additional group of adult male ICR mice aged 12–16 weeks. After a single intravenous administration of $5x10^5$ DiR-labeled hiPSC-MSCs, mice were sacrificed on day 1 or 7. After sacrifice, splenocytes, livers, hearts, kidneys and lungs were harvested for epi-fluorsecent imaging using a IVIS® Spectrum optical imaging system under field of view (FOV) 3.9x3.9 cm (PerkinElmer, Inc. MA). The injected DiR labeled cells were visualized under 750 nm/800 nm excitation/emission wavelength; and fluorescent intensity represented by radiant efficiency.

Next, the optimal timing of systemic hiPSC-MSCs preconditioning before direct intramyocardial transplantation was determined at three different time points. After a single intravenous injection of $5x10^5$ hiPSC-MSCs, mice were sacrificed on (1) day 3 (Day 3 group, n=3); (2) day 7 (Day 7 group, n=3); or (3) day 11 (Day 11 group, n=3). After sacrifice, fresh splenocytes were harvested from all animals for flow cytometry analysis to detect changes to Treg, $CD4^+$, $CD8^+$ and NK cells in the spleen.

*In-vitro* co-culturing of $CD4^+$ splenocytes with hiPSC-MSCs was performed to assess the immunomodulatory effect of hiPSC-MSCs. CD4 positive cells were isolated by labeling the cells with anti CD4-FITC antibody for 30 min on ice and subsequently selected by fluorescence-activated cell sorting (FACS, MoFlo XDP cell sorter, Beckman Coulter's Life Science, Indianas). Then, $5x10^5$ $CD4^+$ splenocytes were co-cultured with $1x10^5$ hiPSC-MSCs for 7 days in transwell permeable supports (multiple well plate, Corning Life Science, MA). Supernatant was collected for cytokine analysis using multiplex cytokine quantitation array as per the manufacturer's instructions (Ray Biotech, Norcross, GA).

**References:**


1. Lian Q, Zhang Y, Zhang J, Zhang HK, Wu X, Zhang Y, et al. Functional mesenchymal stem cells derived from human induced pluripotent stem cells attenuate limb ischemia in mice. Circulation. 2010; 121: 1113-23.
2. Lian Q, Zhang Y, Liang X, Gao F, Tse HF. Directed Differentiation of Human-Induced Pluripotent Stem Cells to Mesenchymal Stem Cells. Methods Mol Biol. 2016; 1416: 289-98.
3. Liao S, Zhang Y, Ting S, Zhen Z, Luo F, Zhu Z, et al. Potent immunomodulation and angiogenic effects of mesenchymal stem cells versus cardiomyocytes derived from pluripotent stem cells for treatment of heart failure. Stem Cell Res Ther. 2019; 10: 78.
4. Sun YQ, Zhang Y, Li X, Deng MX, Gao WX, Yao Y, et al. Insensitivity of human iPS cells-derived mesenchymal stem cells to interferon-γ-induced HLA expression potentiates repair efficiency of hind limb ischemia in immune humanized NOD SCID Gamma mice. Stem Cells. 2015; 33: 3452-67.
5. Lee YK, Lau YM, Cai ZJ, Lai WH, Wong LY, Tse HF, et al. Modeling Treatment Response for Lamin A/C Related Dilated Cardiomyopathy in Human Induced Pluripotent Stem Cells. J Am Heart Assoc. 2017; 6: e005677.
6. Ng KM, Mok PY, Butler AW, Ho JC, Choi SW, Lee YK, et al. Amelioration of X-Linked Related Autophagy Failure in Danon Disease With DNA Methylation Inhibitor. Circulation. 2016; 134: 1373-89.
7. Xu J-Y, Lee Y-K, Ran X, Liao S-Y, Yang J, Au K-W, et al. Generation of Induced Cardiospheres via Reprogramming of Skin Fibroblasts for Myocardial Regeneration. Stem Cells. 2016; 34: 2693-706.
8. Nikodemova M and Watters JJ. Outbred ICR/CD1 mice display more severe neuroinflammation mediated by microglial TLR4/CD14 activation than inbred C57Bl/6 mice. Neuroscience. 2011; 190: 67-74.
9. Liao SY, Liu Y, Siu CW, Zhang Y, Lai WH, Au KW, et al. Proarrhythmic risk of embryonic stem cell-derived cardiomyocyte transplantation in infarcted myocardium. Heart Rhythm. 2010; 7: 1852-9.
10. Casiraghi F, Azzollini N, Cassis P, Imberti B, Morigi M, Cugini D, et al. Pretransplant Infusion of Mesenchymal Stem Cells Prolongs the Survival of a

Semiallogeneic Heart Transplant through the Generation of Regulatory T Cells. J Immunol. 2008; 181: 3933-46.
11. Casiraghi F, Azzollini N, Todeschini M, Cavinato RA, Cassis P, Solini S, et al. Localization of Mesenchymal Stromal Cells Dictates Their Immune or Proinflammatory Effects in Kidney Transplantation. Am J Transplant. 2012; 12: 2373-83.
12. Trac D, Maxwell JT, Brown ME, Xu C and Davis ME. Aggregation of Child Cardiac Progenitor Cells into Spheres Activates Notch Signaling and Improves Treatment of Right Ventricular Heart Failure. Circ Res. 2019; 124: 526-38.
13. Lai W-H, Ho JCY, Chan Y-C, Ng JHL, Au K-W, Wong L-Y, et al. Attenuation of hind-limb ischemia in mice with endothelial-like cells derived from different sources of human stem cells. PLoS One. 2013; 8: e57876.
14. Lv K, Li Q, Zhang L, Wang Y, Zhong Z, Zhao J, et al. Incorporation of small extracellular vesicles in sodium alginate hydrogel as a novel therapeutic strategy for myocardial infarction. Theranostics. 2019; 9: 7403-16.
15. Mills CD, Kincaid K, Alt JM, Heilman MJ and Hill AM. M-1/M-2 Macrophages and the Th1/Th2 Paradigm. J Immunol. 2000; 164: 6166-73.
16. Feher K, Kirsch J, Radbruch A, Chang H-D and Kaiser T. Cell population identification using fluorescence-minus-one controls with a one-class classifying algorithm. Bioinformatics. 2014; 30: 3372-8.
17. Zhang Y, Yu Z, Jiang D, Liang X, Liao S, Zhang Z, et al. iPSC-MSCs with High Intrinsic MIRO1 and Sensitivity to TNF-α Yield Efficacious Mitochondrial Transfer to Rescue Anthracycline-Induced Cardiomyopathy. Stem Cell Reports. 2016; 7: 749-63.
18. Potluri K, Mahas A, Kent MN, Naik S and Markey M. Genomic DNA extraction methods using formalin-fixed paraffin-embedded tissue. Anal Biochem. 2015; 486: 17-23.

## Supplemental figures and figure legends.

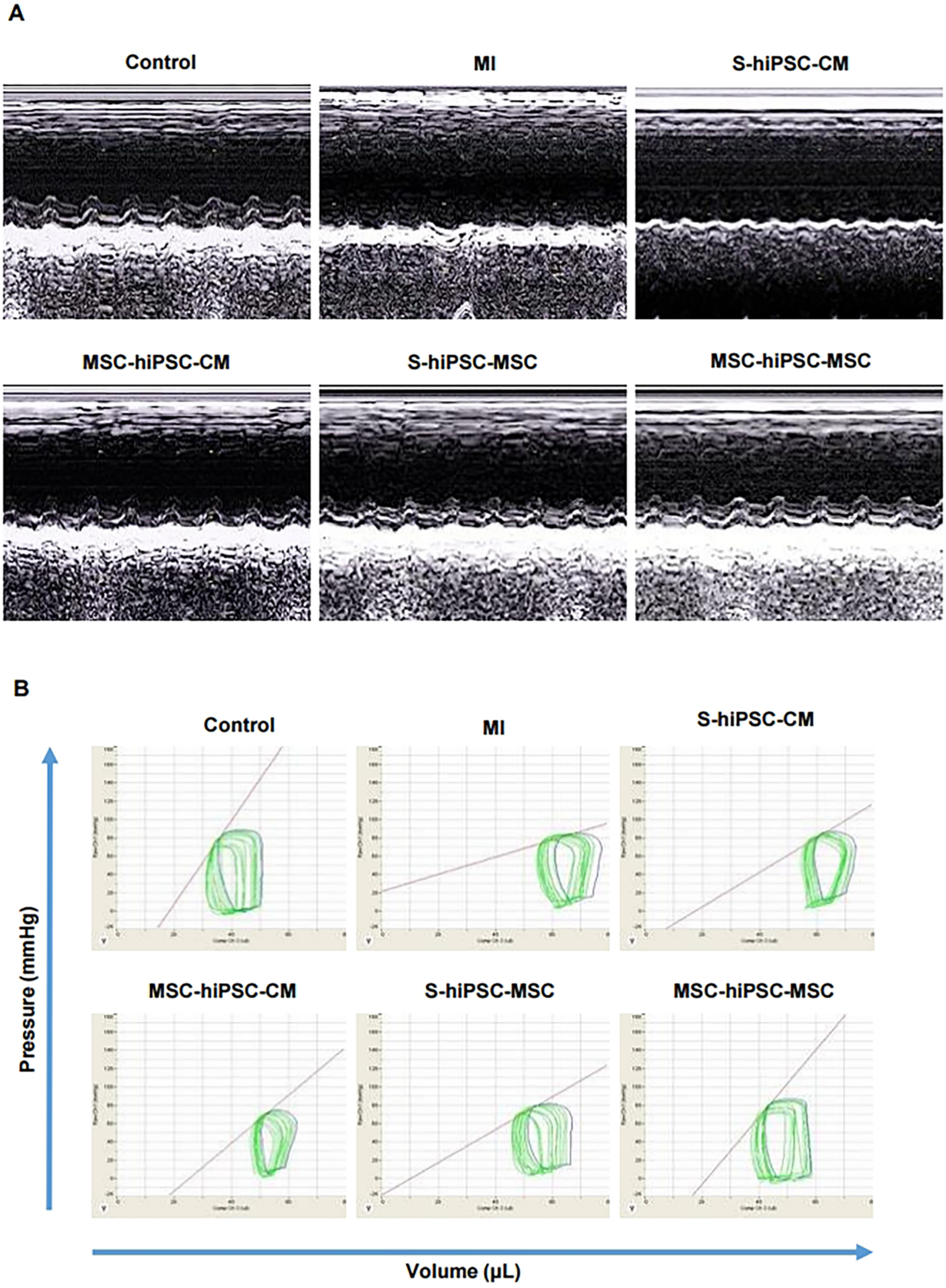


**Figure S1.** Effects of intravenous pre-transplantation systemic administration of hiPSC-MSCs on cardiac function after intramyocardially transplanted hiPSC-CMs or hiPSC-MSCs.

Transthoracic echocardiogram images of left ventricular (LV) ejection fraction (LVEF), fractional shortening (FS) and LV dimension at week 4 **(A)**. Invasive hemodynamic assessment of pressure-volume loop images of LV maximal positive pressure derivative (+dP/dt$_{max}$) and end systolic pressure-volume relationship (ESPVR) at week 4 **(B)**.

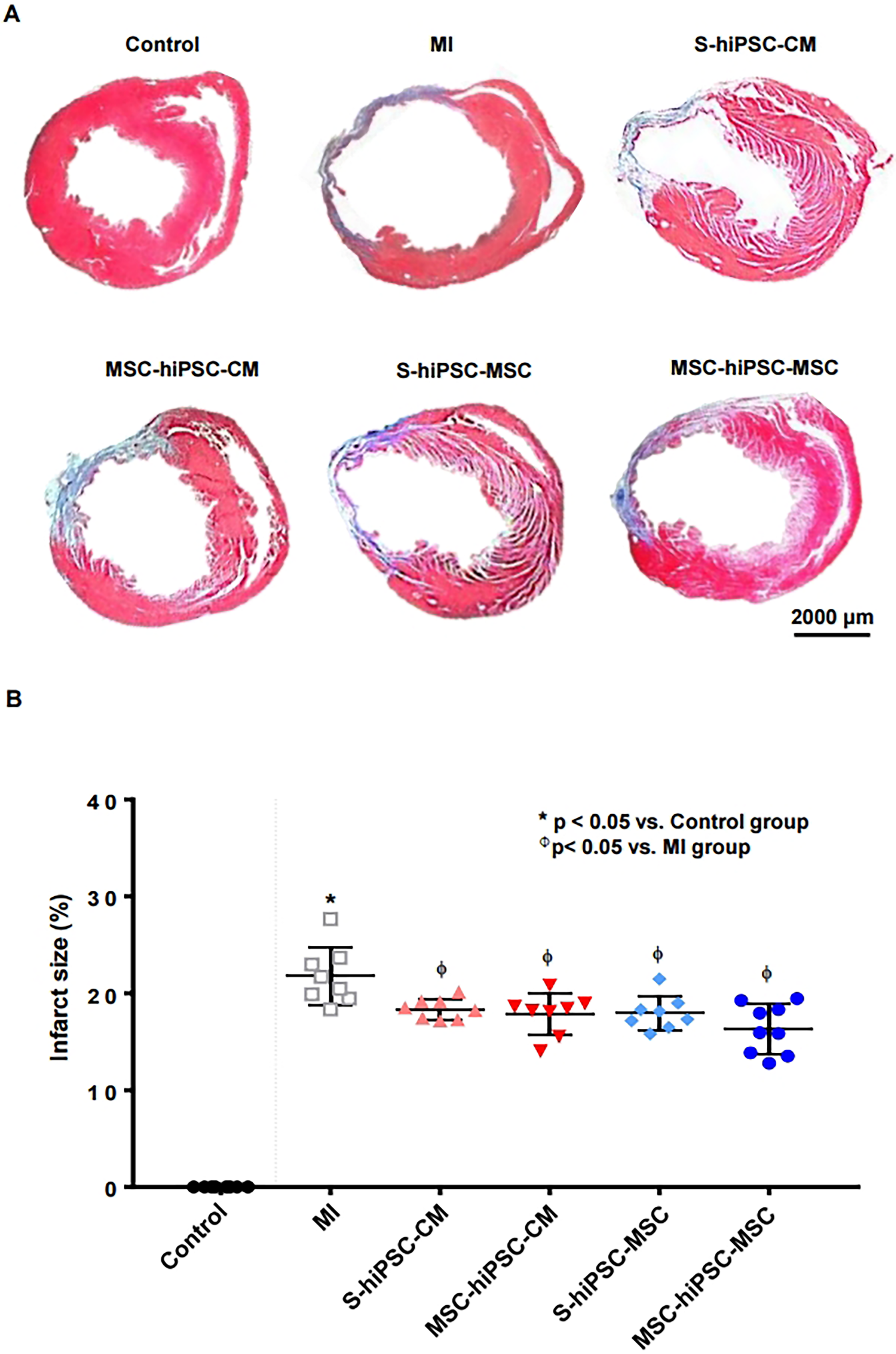


**Figure S2.** Effects of intravenous pre-transplantation systemic administration of hiPSC-MSCs on infarct size after intramyocardially transplanted hiPSC-CMs or

hiPSC-MSCs.

To define infarct size, Masson's trichrome staining of cardiac tissue sections was performed in all experimental mice 4 weeks after induction of myocardial infarction (MI) **(A)**. MI area was represented by fibrotic tissue (blue) and cardiac tissue was stained red. Quantification of infarct size was evaluated by the area of fibrotic tissue compared with the area of whole LV tissue. Pre-transplantation systemic administration of hiPSC-MSCs in both MSC-hiPSC-CM and MSC-hiPSC-MSC groups did not further decrease infarct size compared with the S-hiPSC-CM and S-hiPSC-MSC groups **(B)**.

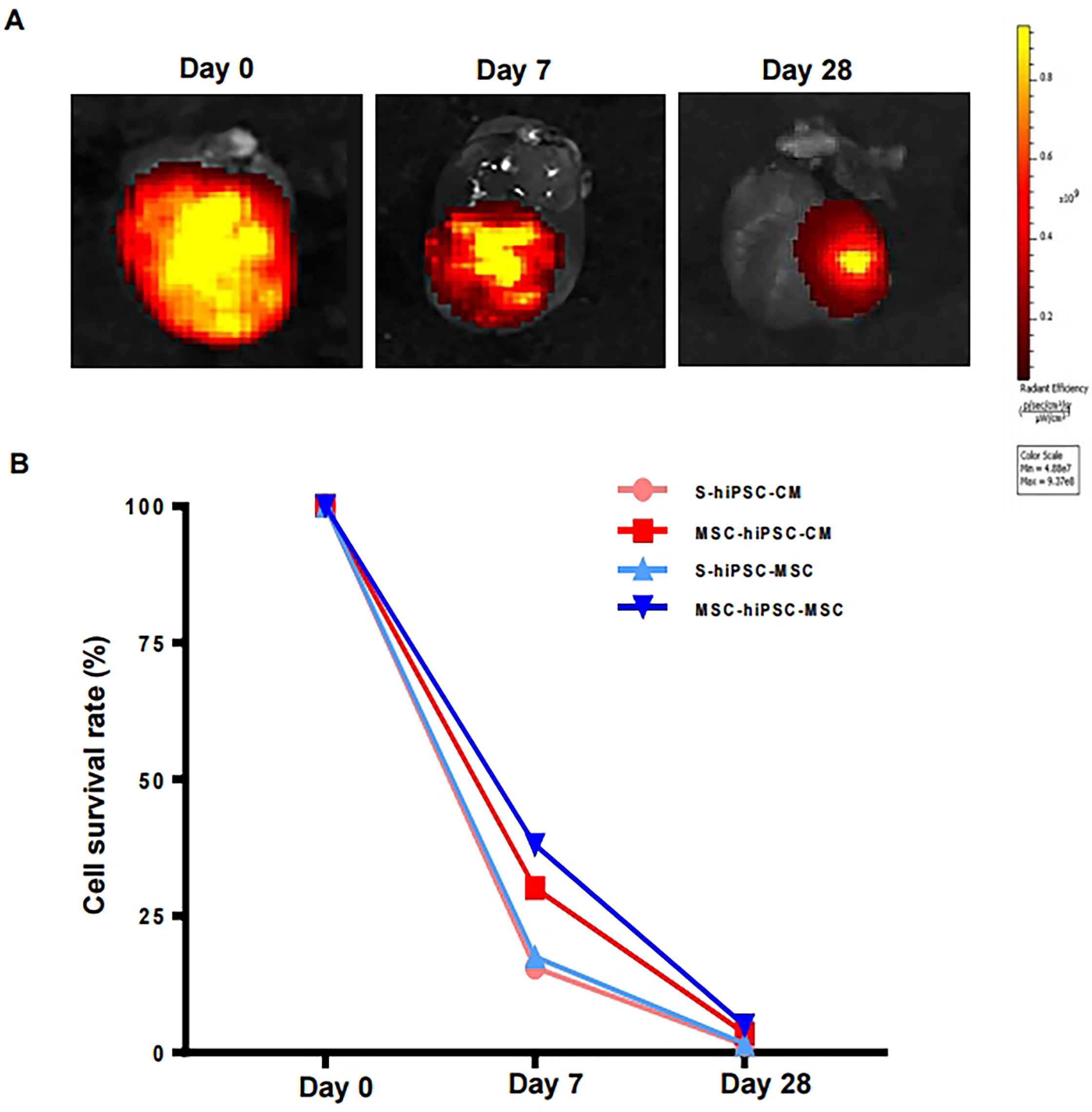


| | S-hiPSC-CM | MSC-hiPSC-CM | S-hiPSC-MSC | MSC-hiPSC-MSC |
|---|---|---|---|---|
| Day 7 | 15.51% | 29.15% | 17.54% | 38.01% |
| Day 28 | 1.35% | 3.51% | 1.62% | 4.95% |

**Figure S3.** The survival rate of intramyocardial transplanted hiPSC-CMs or hiPSC-MSCs at day 7 and 28.

The mean of radiant efficiency in each group at day 0, 7 or 28 was calculated. The survival rate of intramyocardial transplanted cells in each group at day 7 or 28 was expressed as the percentage of the mean of radiant efficiency at day 7 or 28 and the mean of radiant efficiency at day 0 **(A)**. The survival rates of intramyocardial transplanted cells at day 7 in S-hiPSC-CM, MSC-hiPSC-CM, S-hiPSC-MSC and

MSC-hiPSC-MSC groups were 15.54%, 30.09%, 17.57% and 38.01%, respectively. The survival rates of intramyocardial transplanted cells at day 28 in S-hiPSC-CM, MSC-hiPSC-CM, S-hiPSC-MSC and MSC-hiPSC-MSC groups were 1.35%, 3.51%, 1.63% and 4.95%, respectively **(B)**.

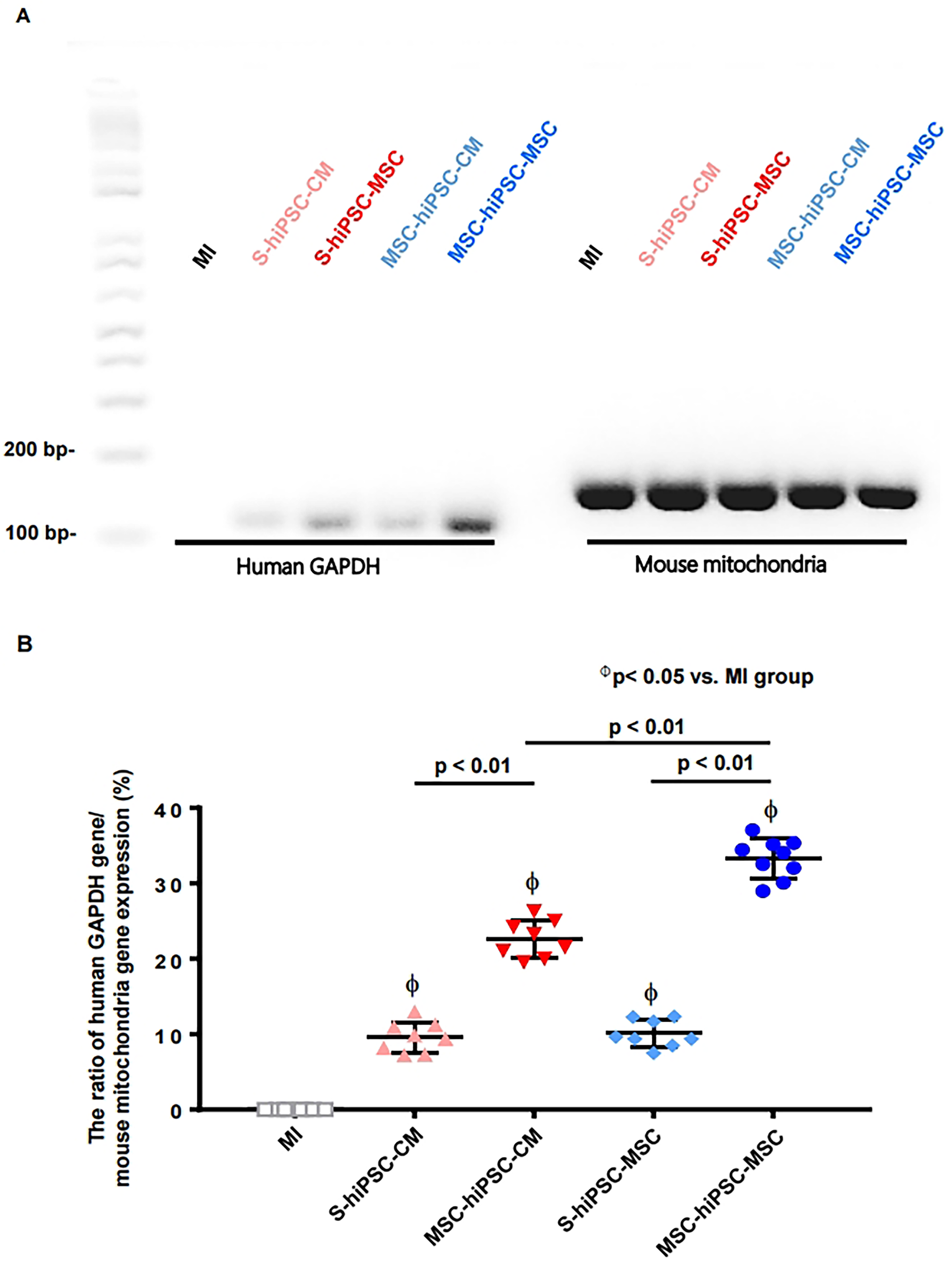


**Figure S4.** Effects of intravenous pre-transplantation systemic administration of hiPSC-MSCs on cell retention after intramyocardially transplanted hiPSC-CMs or hiPSC-MSCs.

Genomic polymerase chain reaction (PCR) of human GAPDH was performed 4 weeks after intramyocardial transplantation to confirm cell retention. Hearts obtained

from the MI group served as negative controls. The expression of human GAPDH gene was detected in all intramyocardial hiPSC-CMs or hiPSC-MSCs transplanted groups with or without pre-transplantation of hiPSC-MSCs. The relative expression of the human GAPDH gene was significantly higher after pre-transplantation systemic administration of hiPSC-MSCs in both MSC-hiPSC-CM and MSC-hiPSC-MSC groups compared with the S-hiPSC-CM and S-hiPSC-MSC groups, respectively ($P<0.01$). Expression of human GAPDH was also significantly higher in the MSC-hiPSC-MSC group compared with the MSC-hiPSC-CM group ($P<0.01$).

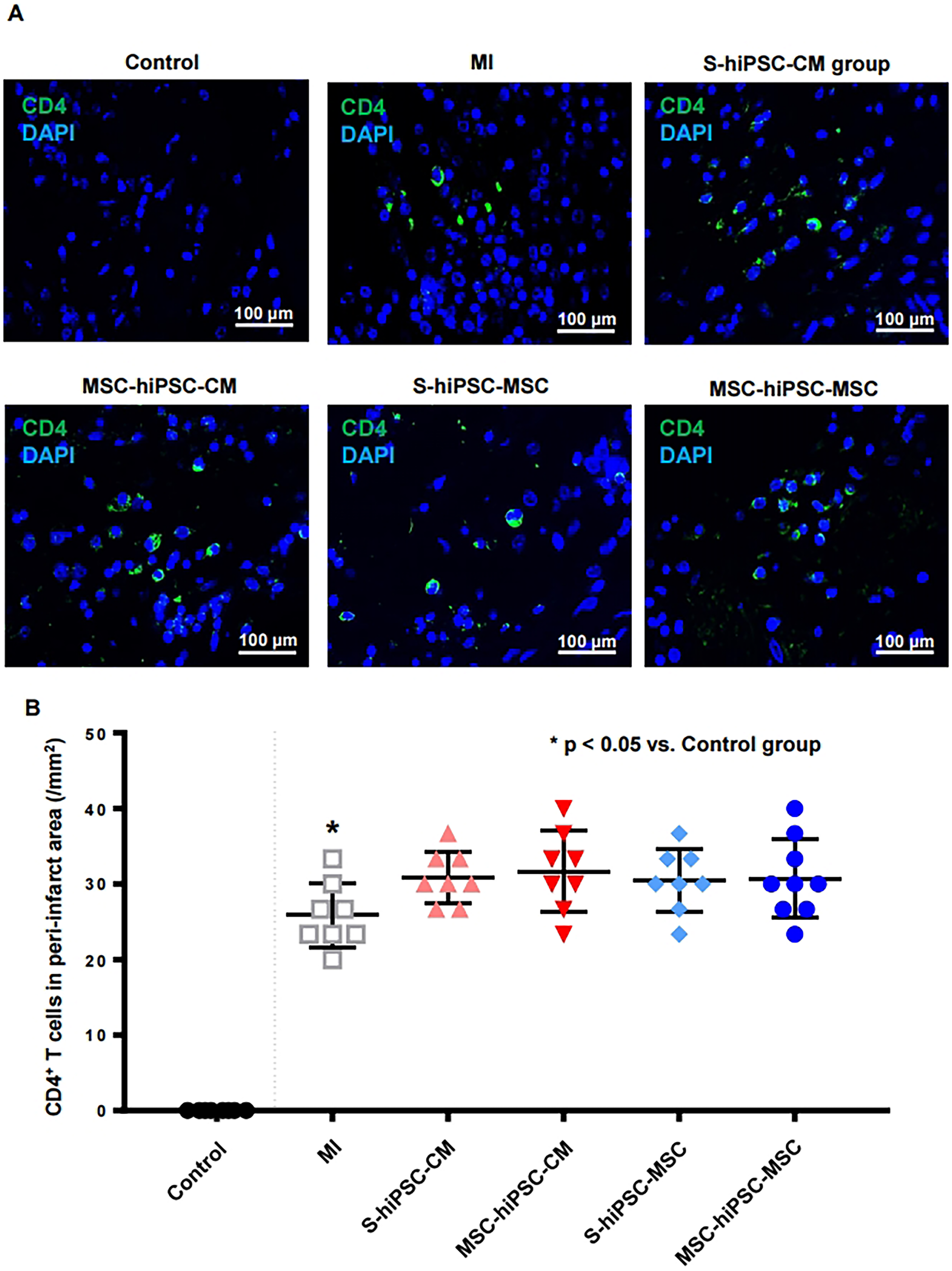


**Figure S5.** Effects of intravenous pre-transplantation systemic administration of hiPSC-MSCs on $CD4^+$ cells in the peri-infarct area.

Immunostaining of anti-mouse CD4 was performed in all experimental mice 4 weeks after myocardial infarction induction to define $CD4^+$ T cell infiltration in the peri-infarct area **(A)**. Intramyocardial transplantation of hiPSC-CMs or hiPSC-MSCs with or

without pre-transplantation systemic administration did not alter infiltration of $CD4^+$ T cells, compared with MI group **(B)**.

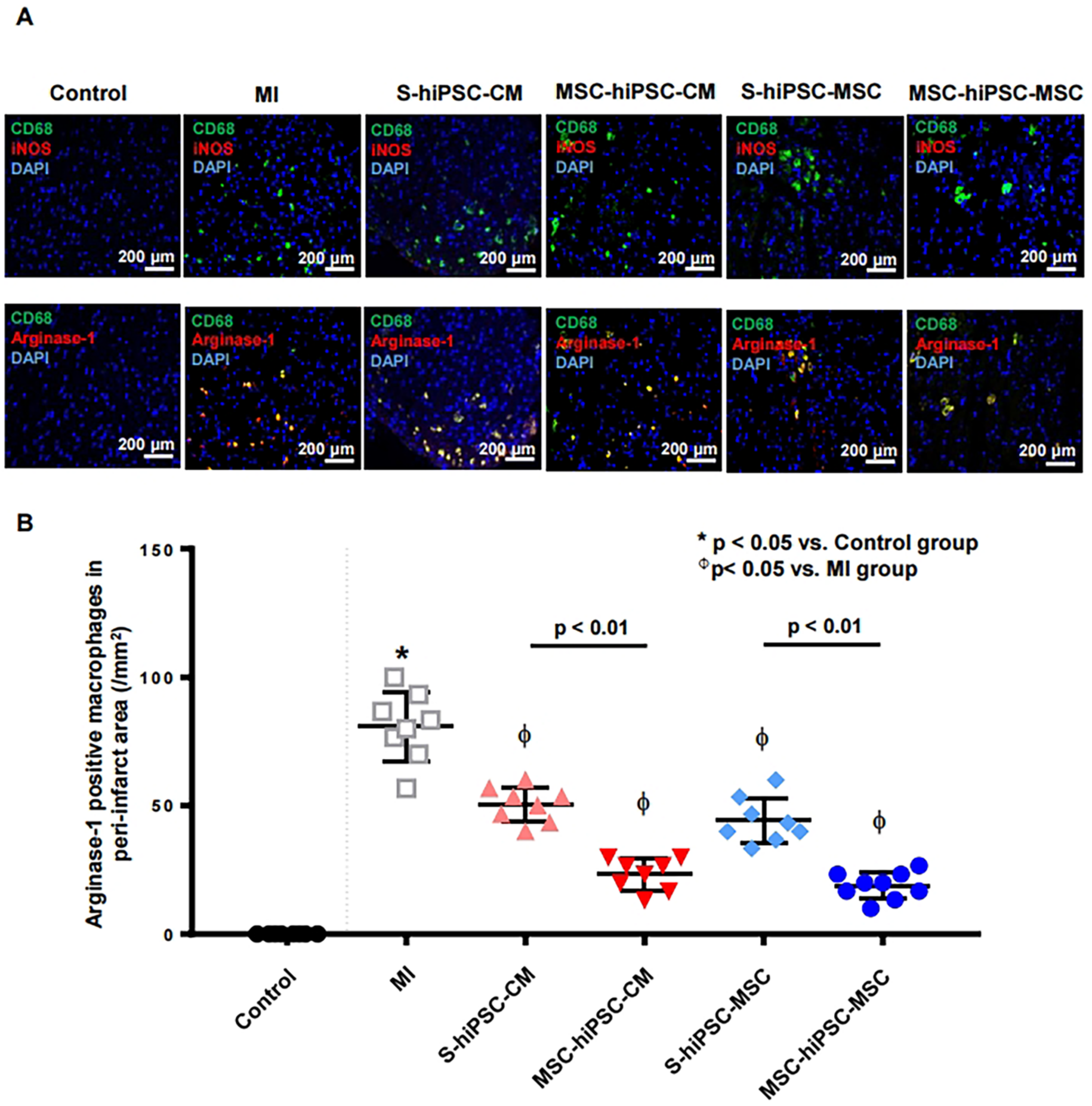


**Figure S6.** Effects of intravenous pre-transplantation systemic administration of hiPSC-MSCs on M1 and M2 macrophages in the peri-infarct area.

Immunostaining of anti-mouse iNOS or anti-mouse arginase-1 co-stained with anti-mouse CD68 was performed in the heart to define the phenotype of macrophages **(A)**. No iNOS positive macrophages were detected in the peri-infarct area **(A)**. Intramyocardial transplantation of CMs or MSCs in S-hiPSC-CM group or S-hiPSC-MSC group decreased myocardial arginase-1 positive macrophages, compared with the MI group. Moreover, pre-transplantation systemic administration of hiPSC-MSCs in both MSC-hiPSC-CM and MSC-hiPSC-MSC groups further decreased myocardial arginase-1 positive macrophages in the peri-infarct area compared with S-hiPSC-CM group and S-hiPSC-MSC group **(B)**.

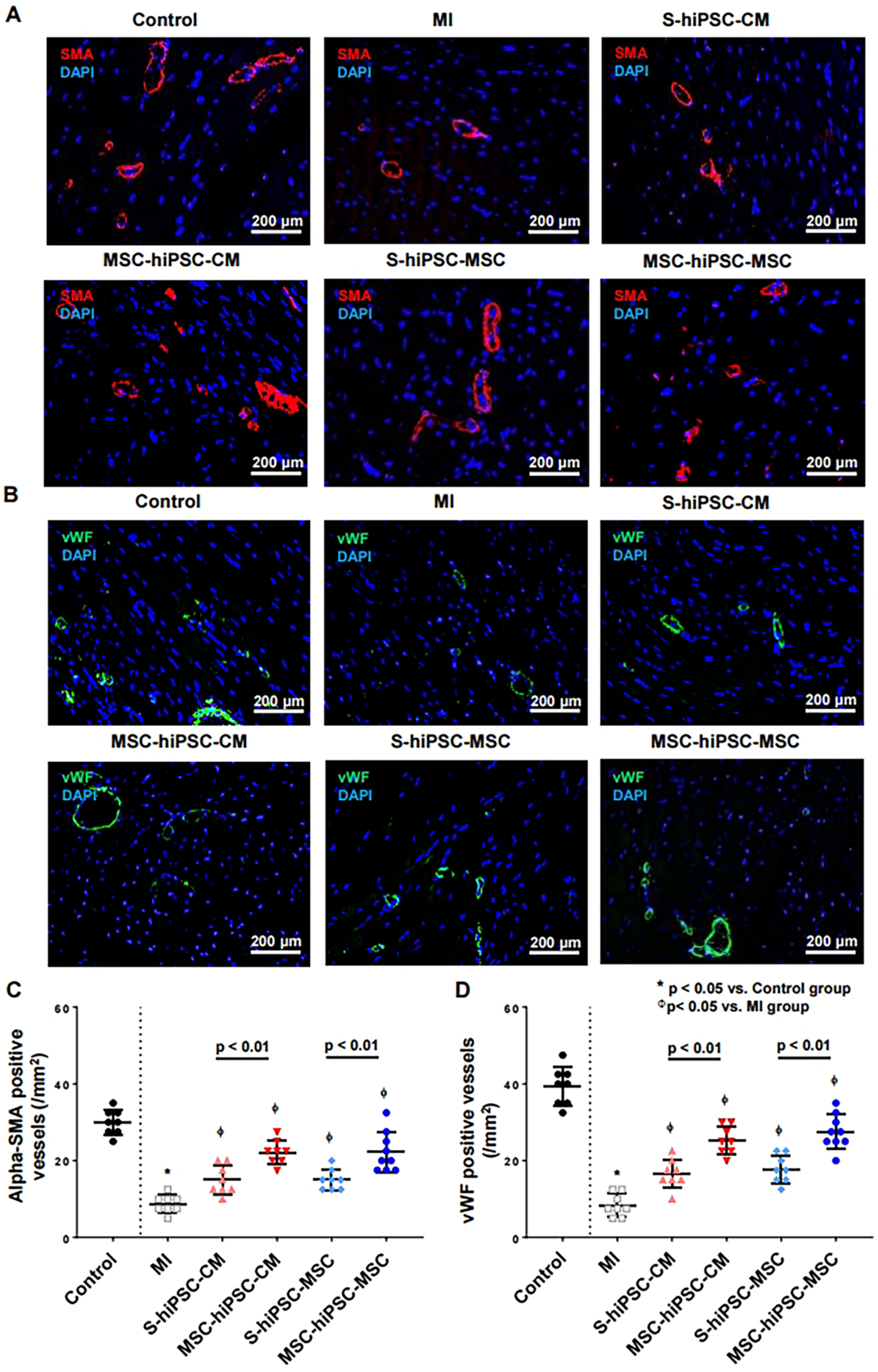


**Figure S7.** Effects of intravenous pre-transplantation systemic administration of

hiPSC-MSCs on neovascularization in the peri-infarct region.

To assess neovascularization in the peri-infarct area, immunofluorescent staining of anti-α-SMA (red) was performed and cell nuclei were counterstained by DAPI (blue) **(A)**. Immunofluorescent staining of anti-vWF (green) was performed and cell nuclei were counterstained by DAPI (blue) **(B)**. The degree of neovascularization was measured by α-SMA positive vessels **(C)** or vWF positive vessels **(D)** under fluorescent microscopy and expressed as count per $mm^2$. Pre-transplantation systemic administration of hiPSC-MSCs in both MSC-hiPSC-CM and MSC-hiPSC-MSC groups increased capillary density compared with the S-hiPSC-CM and S-hiPSC-MSC groups, respectively.

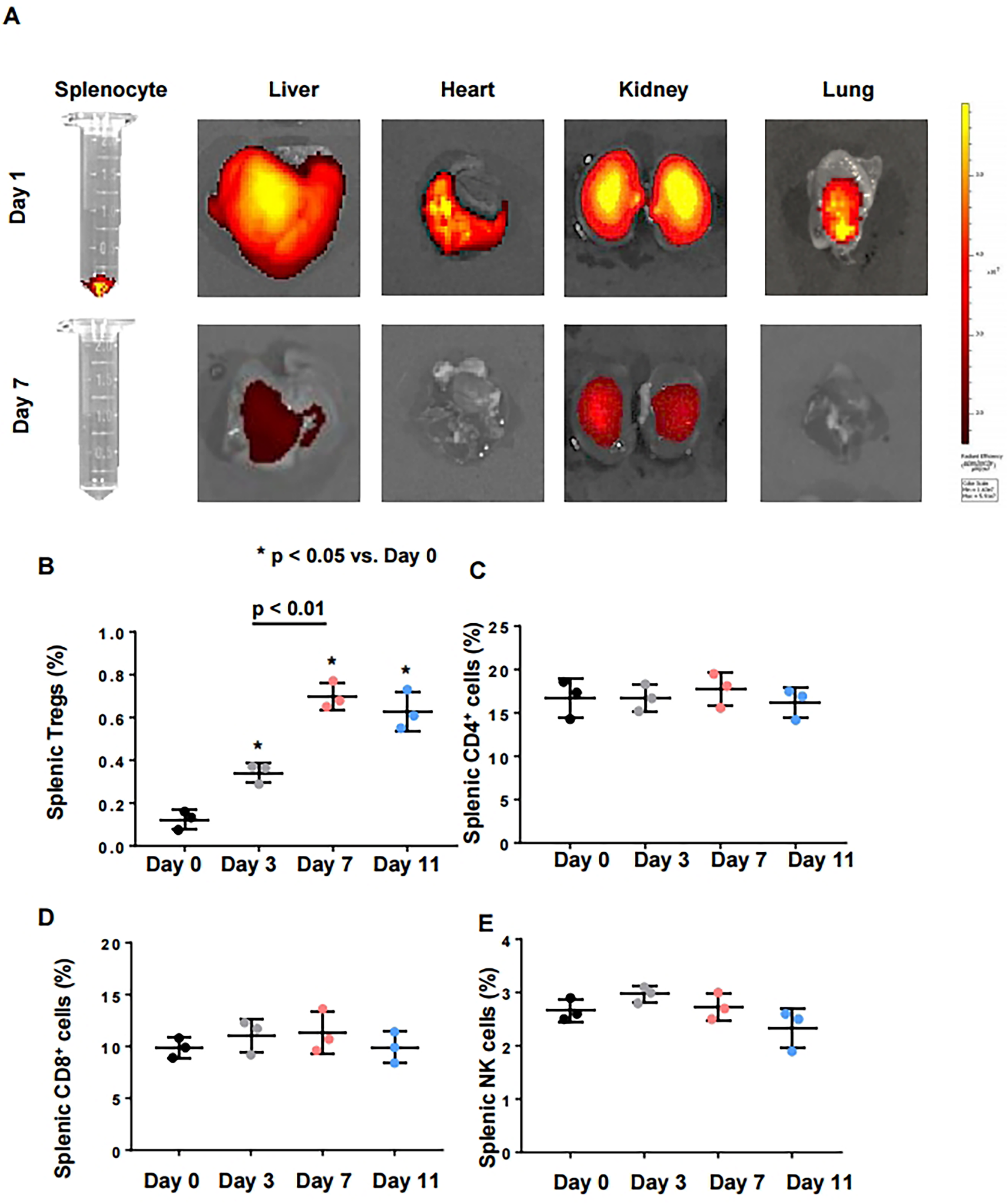


**Figure S8.** The fate of intravenous pre-transplantation systemic administration of hiPSC-MSCs in major organs and effect on splenic immune cells.

Fluorescent imaging of the harvested splenocytes, livers, hearts, kidneys and lungs was performed to evaluate cellular engraftment of the labeled hiPSC-MSCs at 1 and 7 days after a single intravenous injection. Most cells had been cleared from the major organs, especially the hearts and lungs, 7 days after injection (**A**). After a single intravenous injection of hiPSC-MSCs, splenocytes were freshly isolated and

analyzed by flow cytometry on day 3, 7 and 11 to measure the splenic Tregs (**B**), $CD4^+$(**C**), $CD8^+$ (**D**) and NK (**E**) population. Splenic Tregs reached their peak level on day 7 after a single intravenous injection of hiPSC-MSCs, while no significant change was observed in the $CD4^+$, $CD8^+$ or NK population.

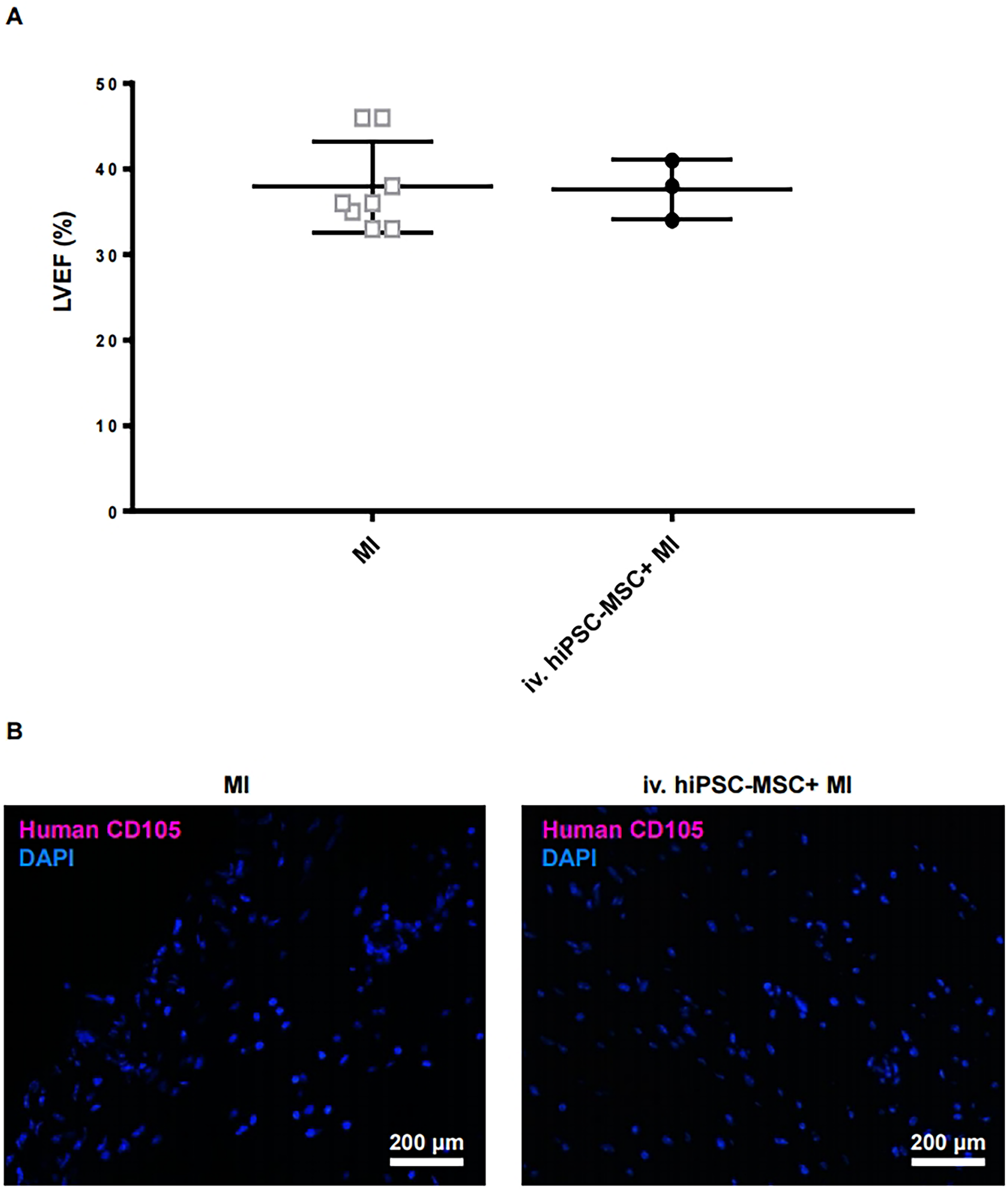


**Figure S9.** Intravenous pre-transplantation systemic administration of hiPSC-MSCs alone resulted in no functional improvement or transplanted cell retention in the infarcted heart.

Intravenous administration of $5x10^5$ hiPSC-MSCs one week before induction of myocardial infarction (MI) had no significant therapeutic effect on LV function compared with the MI group (**A**). Immunostaining with anti-human CD105 was performed to detect the presence of intravenously transplanted hiPSC-MSCs in the infarcted heart. No transplanted hiPSC-MSCs were detected in the infarcted heart

(**B**).

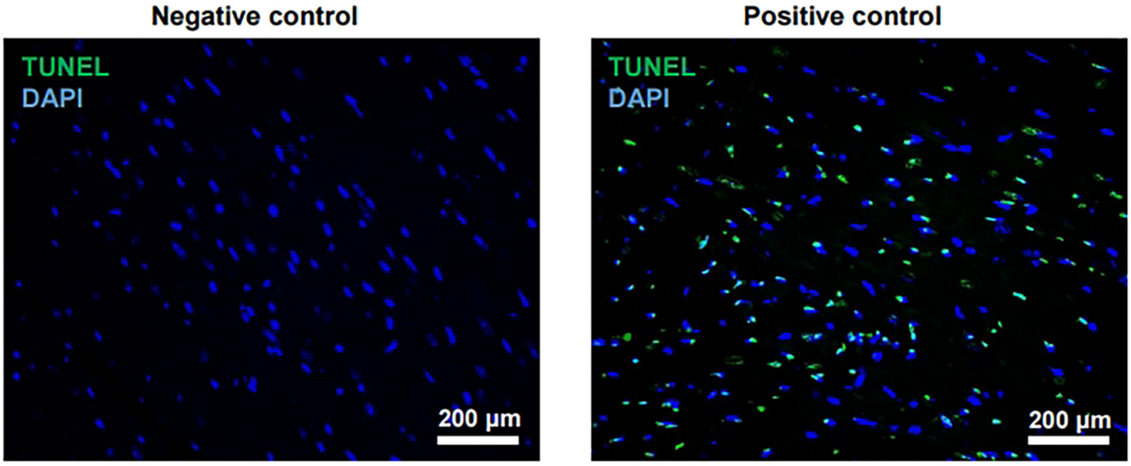


**Figure S10.** The positive and negative controls of TdT-mediated dUTP Nick-End Labeling (TUNEL) staining.
